# Enzymes for Detoxification of Various Mycotoxins: Origins and Mechanisms of Catalytic Action

**DOI:** 10.3390/molecules24132362

**Published:** 2019-06-26

**Authors:** Ilya Lyagin, Elena Efremenko

**Affiliations:** 1 Faculty of Chemistry, Lomonosov Moscow State University, Moscow 119991, Russia; 2 Emanuel Institute of Biochemical Physics, RAS, Moscow 119334, Russia

**Keywords:** enzyme, mycotoxin, conversion, biochemical mechanism, molecular modeling, origins, detoxification, antidote

## Abstract

Mycotoxins are highly dangerous natural compounds produced by various fungi. Enzymatic transformation seems to be the most promising method for detoxification of mycotoxins. This review summarizes current information on enzymes of different classes to convert various mycotoxins. An in-depth analysis of 11 key enzyme mechanisms towards dozens of major mycotoxins was realized. Additionally, molecular docking of mycotoxins to enzymes’ active centers was carried out to clarify some of these catalytic mechanisms. Analyzing protein homologues from various organisms (plants, animals, fungi, and bacteria), the prevalence and availability of natural sources of active biocatalysts with a high practical potential is discussed. The importance of multifunctional enzyme combinations for detoxification of mycotoxins is posed.

## 1. Introduction

**Figure 1:**
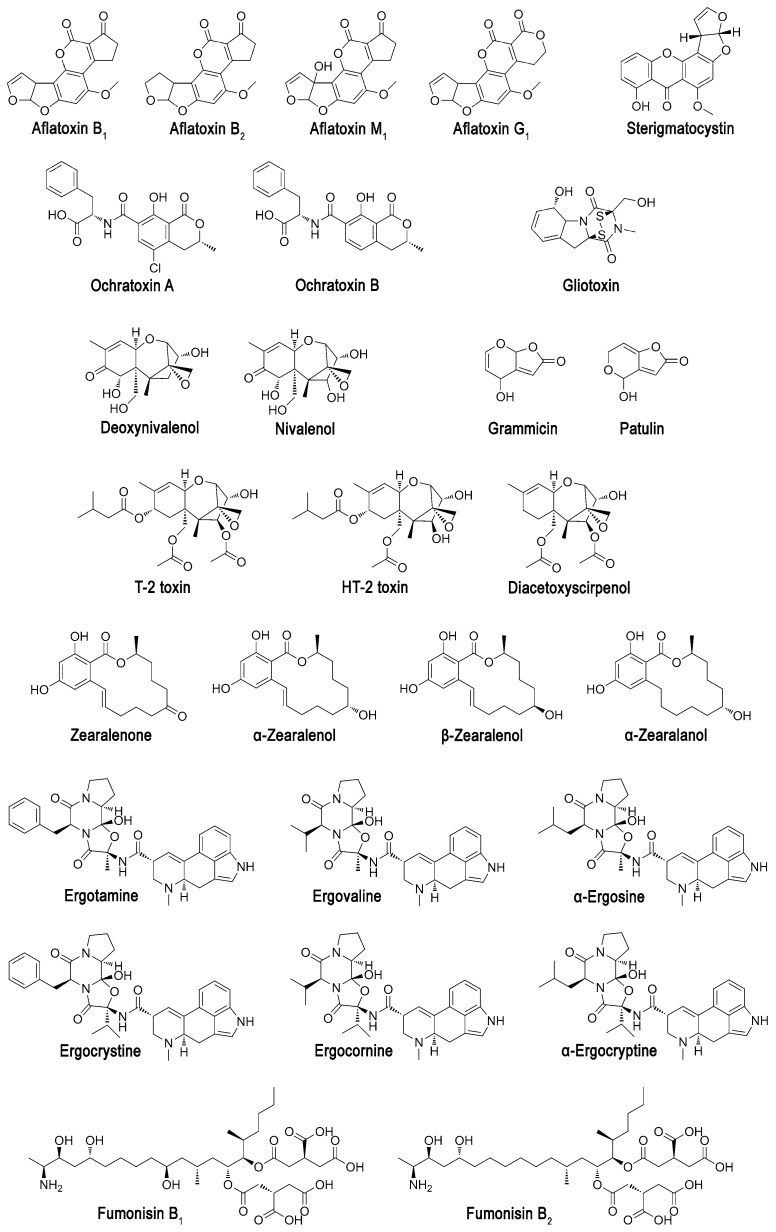
Chemical structures of some mycotoxins.

To date, more than 100 thousand fungal species have been identified and systematized. According to recent moderate estimates, the number of species could reach up to four million [1]. Owing to their active enzymes, fungi can survive under various conditions and can advance using a wide spectrum of substrates. To be competitive with other organisms (including other fungi) for habitat and substrates, numerous fungi especially of genus *Aspergillus*, *Mucor*, *Penicillium*, *Fusarium*, *Alternaria*, *Stachybotrys*, *Trichoderma* synthesize mycotoxins to act as poisons and inhibitors for different biological targets. Mycotoxins have different chemical structure (Figure 1) and toxicity [2], and are usually present in agricultural raw materials, food products, and feedstuffs, thus being a real threat for health of humans and animals [3].

Chronic intoxication of farm animals decreases an overall agricultural productivity [4], and contamination of food and raw materials by mycotoxins results in additional costs. So, decontamination of mycotoxins from various products is a worldwide problem, both scientifically and practically. Physical chemical methods of mycotoxins removal are studied by many researchers [5]. It has been shown that the mycotoxins can be eliminated by physical (thermolysis, radiation treatment, low-temperature plasma, etc.), chemical (oxidation, reduction, hydrolysis, alcoholysis, ad(b)sorption, etc.), and biological methods (similar to chemical methods but by biological agents) [6]. Moreover, only two methods of all the variety of detoxification approaches already known and being developed are permitted now: chemical hydrolysis (with ammonium or sodium hydroxide) and biological detoxification (with feed additive Mycofix^®^ BIOMIN GmbH, etc.). Nevertheless, both of them are allowed in a very limited number of countries for few issues, since these methods have several significant disadvantages—for example, contamination of raw materials with chemicals and/or products of side reactions (they typically have their own toxicity), and decreasing of food value as a result of chemical and biological processes; etc. At the same time, application of commercial sorbents and feed additives may be ineffective too for the mycotoxin removal from the gastrointestinal tract [7,8]. 

Enzymatic detoxification of mycotoxins is devoid of many of these shortcomings, combines the features of chemical and biological treatment and synergizes them. It results in high efficiency and specificity of action; in possible application under mild conditions; in ordinary lack of their own toxicity for organisms, subsequently consuming the processed raw materials. Moreover, enzymes like all catalysts can be used in non-stoichiometric ratios with mycotoxins. Even there may be no loss of aesthetic appearance or food quality of the treated materials. Recently there has been a particularly intense interest in studies of mycotoxin-modifying enzymes [9]. A lot of structural and catalytical data about various enzymes modifying mycotoxins has been accumulated enough, but only a few reviews have been published to date to systematize them [10]. Here, we tried to look deeper into the (bio)chemistry of enzymatic processes, compensating some of existing informational gaps with computer simulation. That is, a lot of enzyme structures are unknown; or are known but don’t contain within their active centers a substrate, reaction product or substrate analogue (inhibitor). Both cases would not allow someone to unambiguously conclude the catalytic mechanism. Molecular modeling (in particular, protein folding and molecular docking) can be applied as powerful tools to solve such problems [11,12], and were used here.

In addition, fungi are known to synthesize and usually secrete several mycotoxins to affect the widest possible spectrum of biological targets. So, practically it is necessary to detoxify mycotoxin mixtures [13], and therefore, preparations containing several efficient enzymes should be developed. Both thorough analysis of enzyme properties and good understanding of catalytic processes are required to accurately select proper enzymes for such combinations. 

Thus, the purpose of the article is a review of mechanisms of mycotoxin-modifying enzymes that have been studied for the last 10–15 years. It seems interesting and useful to compare results of physical chemical investigations of various enzymes from different scientific groups, to reveal general trends and limitations in mycotoxin transformation, to estimate perspective biotechnological applications of enzymes for mycotoxin detoxification, and to summarize information about biological sources for potential isolation of such enzymes.

## 2. Aflatoxins

Aflatoxin B_1_, (6a*R*,9a*S*)-2,3,6a,9a-tetrahydro-4-methoxy-1*H*,11*H*-cyclopenta[c]furo[3′,2′:4,5] furo[2,3-h][1]benzopyran-1,11-dione, is one of the mostly known and thoroughly investigated mycotoxin. Generally, aflatoxins are the most dangerous mycotoxins due to their genotoxicity and cancerogenity [14]. 

**Figure 2:**
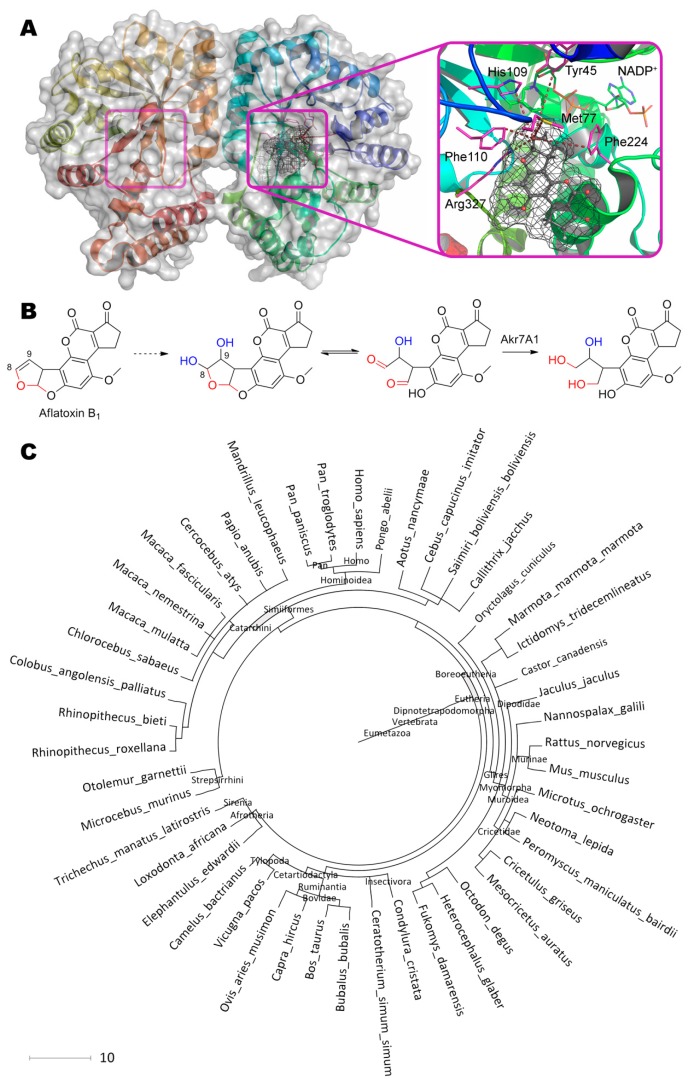
(**A**) Structure of aflatoxin dialdehyde reductase AKR7A1 (PDB 1gve) containing aflatoxin B_1_ in its active center, and (**B**) scheme of substrate conversion with AKR7A1. Position and geometry of substrate binding was determined using molecular docking with Autodock Vina as described [15] (see Appendix A for details). Molecular surface of substrate was calculated using Gamess-US as described [16] and is shown as mesh. Within reaction scheme, OH-groups marked with blue are introduced by cytochromes, and oxygens marked with red are modified by AKR7A1. (**C**) Phylogenetic tree of organisms possessing homologous enzymes found with BLAST.

Aflatoxin dialdehyde aldo-keto reductase AKR7A1 (Figure 2 [15,16], Table 1 [17,18,19,20,21,22,23,24,25,26,27,28,29,30,31,32,33,34,35,36,37,38,39,40,41,42,43,44,45,46,47,48,49,50,51,52,53,54,55,56,57,58]) was isolated from rat liver, and its structure in complex with NADP^+^ was solved [59]. This enzyme is assumed to reduce both aldehyde groups of dialdehyde of hydroxy aflatoxin B_2a_, forming from 8,9-dihydrodiol aflatoxin B_1_, resulted in dihydroxy derivative. Cytochromes are capable to convert aflatoxin B_1_ into 8,9-dihydrodiol derivative [60], and are usually used within mammalians as detoxifying agents of multiple toxic compounds, including mycotoxins [2]. 

The crystallographic structure solved does not contain a substrate; however, molecular docking allows revealing possible poses of binding even aflatoxin B_1_ itself to the active center of AKR7A1. Aflatoxin B_1_ is oriented by the furofuran heterocycle towards catalytically important Tyr45 (7.0 Å) and His109 (4.9 Å) in full compliance with the well-known mechanism of AKR7A1 [59]. Also, residues Met77 (4.2 Å), Phe110 (3.4 Å), Phe224 (5.2 Å) and Arg327 (3.4 Å) are in a close proximity to this heterocycle. Typically, residues Asp and Lys are also required for catalysis to transfer proton from/to Tyr [61]. The most appropriate residues within AKR7A1 seem to be Asp40 and Lys73. 

Homologue of AKR7A1 from human, AKR7A3, has almost 2-times better activity and its pH-optimum shifts to neutral pH [19]. Having an 81% identity and the same catalytic tetrad Tyr–His–Lys–Asp, it contains several point mutations of amino acids forming active center cavity or located closely. Namely, replacements Ala75Ile, Phe110Met and Pro223Thr appear to enlarge this cavity and/or its flexibility, while replacements Ala71Asp and Lys80Asn could shift balance of charged groups in microenvironment of active center. 

Interestingly, another homologue AKR7A5 (80% identity) from mouse has the same pH-optimum as AKR7A1 and improved activity at the expense of specificity [20,21]. The most constitutive replacements are Ala46Cys, Ala75Asn, Met77Trp, Phe78Glu, Phe110Ala, and Pro223Asn. It is noteworthy to add that both AKR7A3 and AKR7A5 have similar replacement Asn47Glu and Asn47Asp, respectively. Indeed, point mutation Met77Trp is the most effective way to shift enzyme specificity [62]. Though both Phe110 and Phe224 could be useful also to exchange specificity for activity and vice versa [63]. 

Curiously enough there are multiple pathways of aflatoxins’ degradation in plants [64,65,66], but there is no furofuran ring opening which is essential for aflatoxins’ tumorigenicity [67]. While enzyme homologues to AKR7A1 are widely spread among mammalians of subclass *Theria* (totally more than 46 species), including humans and other primates. Different AKR7As are known to have a wide activity towards carbonyls [19,20,21] with some preference to 9,10-phenanthrenequinone, 2-carboxybenzaldehyde and succinic semialdehyde, and are acting as detoxification agents. So, RNAs related to AKR7A biosynthesis are located dominantly in the gastrointestinal tract, liver, and kidney of humans [19], and active AKR7As itself could be found even in the brain [68]. Anyway, there are a lot of basic isoforms of enzyme to choose from for applying them in practice and for improving their efficiency towards aflatoxins.

Alternatively, dialdehyde aflatoxin B_2a_ can form arginine adducts, thereby modifying, for example, proteins and, thus, being detoxified [69]. Albeit such mechanism to reduce the toxicity of mycotoxins is not due to enzyme action, it may be of some interest to both toxicologists and analytical chemists.

Laccase from *Trametes versicolor* is capable to detoxify aflatoxin B_1_ [22]. Increased selectivity of enzyme towards aflatoxin B_1_ is observed under conditions (pH 3.5, 55 °C) differing from the optimal ones. Since there is no mutagen activity of detoxification product, this enzyme like cytochromes is acting on the toxigenic double bond of furofuran ring (Figure 2B). Thus, researchers could look for 8,9-dihydrodiol aflatoxin B_1_ as reaction product of this enzyme.

Such laccase from *Trametes versicolor* was immobilized on a porous polyvinylidene fluoride membrane covered with polydopamine and polyethyleneimine and was used to degrade aflatoxin B_1_ [70]. However, the efficiency of mycotoxin removal using such biocatalyst did not differ from that of the same membrane without enzyme. That is, the aflatoxin B_1_ removal occurred only due to ad(b)sorption. Obviously, retention time of influent with biocatalyst was insufficient, as concluded by the authors of this study, as well as by other investigators [71], and/or catalytic activity of the immobilized enzyme was too low. The efficiency of such detoxification system can be further improved in many times by introduction of additional redox mediators [72].

Thermolabile oxidoreductase BADE was isolated from bacteria *Bacillus shackletonii* [23]. Although its activity was extremely low, the enzyme had broad and roughly similar substrate specificity towards aflatoxins B_1_, B_2_, and M_1_. Moreover, mechanism should be investigated further.

Manganese peroxidase (MnP) of 40–45 kDa capable to degrade aflatoxin B_1_ was isolated from white rot *Phanerochaete sordida* [73]. This enzyme oxidizes substrate at pH 4.5 to epoxy-derivative (at C8 and C9) like already mentioned cytochromes. Then, the de-epoxidation of this intermediate product occurs in the presence of H_2_O_2_, forming 8,9-dihydrodiol aflatoxin B_1_. Interestingly, this enzyme is used to degrade plant lignin; therefore, theoretically, both processes of oxidative degradation of lignin and detoxification of aflatoxin B_1_ can be combined during processing of plant materials with this enzyme.

Another MnP degrading aflatoxin B_1_ was isolated from basidiomycota *Pleurotus ostreatus* [24]. Its gene contains only 497 bp encoding 165 amino acids with a molar mass of 18 kDa, that significantly contradicts with 42 kDa from electrophoresis data under denaturing conditions. Though the source for such discrepancy is not discussed by the authors somehow, it may indicate either a tremendous experimental error or a considerable post-translational modification of the enzyme (i.e., the molar mass of dimer and trimer could be 36 and 54 kDa, respectively). Authors [74] assumed similar peroxidase from the same *Pleurotus ostreatus* with molar mass of ca. 90 kDa to open the lactone ring according to measurements of biotransformed mycotoxin fluorescence. Other authors [25] went further and supposed (so far without experimental confirmation) that not only the lactone ring but also the coumarin ring can be opened. The commercial horseradish peroxidase showed the highest affinity for aflatoxin B_1_ compared to aflatoxin M_1_. Such enzyme successfully decontaminates milk and beer from the aflatoxins [25] by mechanism still being unknown.

Oxidoreductase BacC from *Bacillus subtilis* also assumed by authors [75] to break a lactone ring of aflatoxin B_1_. However, according to its native activity in bacilysin synthesis [76], this enzyme should rather form an epoxy cycle at the same double bond as aforementioned cytochromes. Consequently, detoxification of aflatoxins by BacC and peroxidases may be similar, i.e., with the formation of the epoxy cycle.

Monomeric aflatoxin oxidase AFO was isolated from honey agarics [26]. This enzyme similarly to cytochromes and MnP oxidizes the furofuran ring at C8 and C9 to an epoxy derivative, which is then oxidized by released H_2_O_2_ to 8,9-dihydrodiol [27]. Interestingly, the nearest structural analogue of aflatoxin B_1_, sterigmatocystin, is also oxidized by the same mechanism. Ion Cu^2+^ is coordinated by residues His443, His448, and Glu501 in the active center [74]. The water molecule, which subsequently replaces the substrate, completes the coordination sphere of the metal ion to tetrahedral. Residues Tyr313 (4.5 Å), Glu444 (4.4 Å) and His562 (4.2 Å) are oriented into the cavity in which the substrate should be, and therefore can participate in its coordination and/or in the catalytic act itself. Interestingly, AFO structure [77], including the geometry of amino acids within active center, has a high degree of homology (except for the closed form of the active center only) with the dipeptidyl peptidase III family. Indeed, the replacement of the Cu^2+^ with Zn^2+^ leads to the appearance of peptidase activity of this oxidoreductase. Subsequent computer calculations by molecular dynamics and docking, together with the analysis of the surface charge distribution, confirmed that the primary AFO activity was peptidase [78], and the enzyme activity towards aflatoxin B_1_ is a non-specific, secondary.

Presumably, the second enzyme ADTZ from the same basidiomycota *Armillariella tabescens* has significantly widened pH-optima 5–8 [28,29]. Its isoelectric point (pI 5.4) was close to pI 5.3 of AFO. Albeit Cu^2+^ decreased enzyme activity by 16%, it can be assumed that ADTZ is a homologue of AFO with a reduced molar mass from another strain of *Armillariella tabescens*.

The enzyme MADE from bacteria *Myxococcus fulvus* fundamentally differed from all aforementioned biocatalysts due to proposed hydrolytic mechanism of its action on aflatoxin B_1_, G_1_, and M_1_ with almost identical efficiency [30]. Chromatographic and mass-spectrometric studies of the reaction products [79] didn’t appear to be reliable in identifying the end product(s). Using IR-spectroscopy, authors associated the peak at 1728 cm^−1^ with a modification of the lactone ring of aflatoxin B_1_. Although it is most likely to be one of the C=O stretching modes in the cyclopentanone conjugated with a lactone ring. Its disappearance is also questionable, and rather it is poorly resolved due to increased influence of the forming acyl group upon opening of the lactone ring. Confirming such ring opening, peak at ca. 1270 cm^−1^ associated with deformational vibrations in the lactone ring as a whole disappeared. Nevertheless, other microorganisms (e.g., bacteria of the genus *Pseudomonas* [80]) are able to open/degrade the lactone ring in aflatoxins; however, these mechanisms are not fully investigated.

A number of flavin-dependent (namely, F_420_H_2_-dependent) oxidoreductases capable to reduce aflatoxins have been isolated from various *Actinomycetales* [31]. Their feature is a reduction of the double bond in the lactone ring of the substrate with subsequent spontaneous hydrolysis of the product and ring opening. This mechanism is possible due to implementation of reduced 8-hydroxy-5-deazoflavin (F_420_H_2_) as a coenzyme, which is susceptible only to two-electron oxidation as opposed to the widespread flavin mononucleotide (in reduced form, FMNH_2_) allowing one and two-electron oxidation. Two enzymes from *Mycobacterium smegmatis* have the highest activity [81].

Computer modeling is emerging and advancing for selection and/or development of enzymes degrading mycotoxins [82]. So, recently molecular dynamics simulations allowed to identify several potential enzymes capable to bind aflatoxin B_1_ [83], namely: trihydroxynaphthalene reductase (1g0o), glycogen synthase kinase GSK-3b (3f88) and serine/threonine kinase Pim-1 (3jya). However, there was no experimental evidence of the activity of these enzymes towards aflatoxin(s) yet.

Docking by molecular dynamics of aflatoxin B_1_ with human cytochrome CYP1A2 revealed its orientation by C8,C9-atoms towards heme catalytic center [32]. Distance between Fe(II) and the closest labile carbon was ca. 4.2 Å. Replacement Thr124Ala leads to 3.7-fold improvement of catalytic efficiency due to 4.4-fold decrease of *K*_m_. While mutations Phe125Ala, Phe226Ala, and Phe260Ala worsen *K*_m_ on orders of magnitude that with undetectable *k*_cat_ results in complete inactivation of first two mutants. Except Thr124, all other residues seem to be responsible for the proper orientation and localization of aflatoxin B_1_ within active site. Polycyclic benzopyrene moiety of aflatoxin B_1_ is possibly of extreme importance. Also authors identified some “impurities” in product mixture which can be aflatoxin M_1_ having OH-group at C9a and forming as by-product of aflatoxin B_1_ oxidation by CYP1A2 (equal to one third of converted substrate [84]).

## 3. Sterigmatocystin

**Figure 3:**
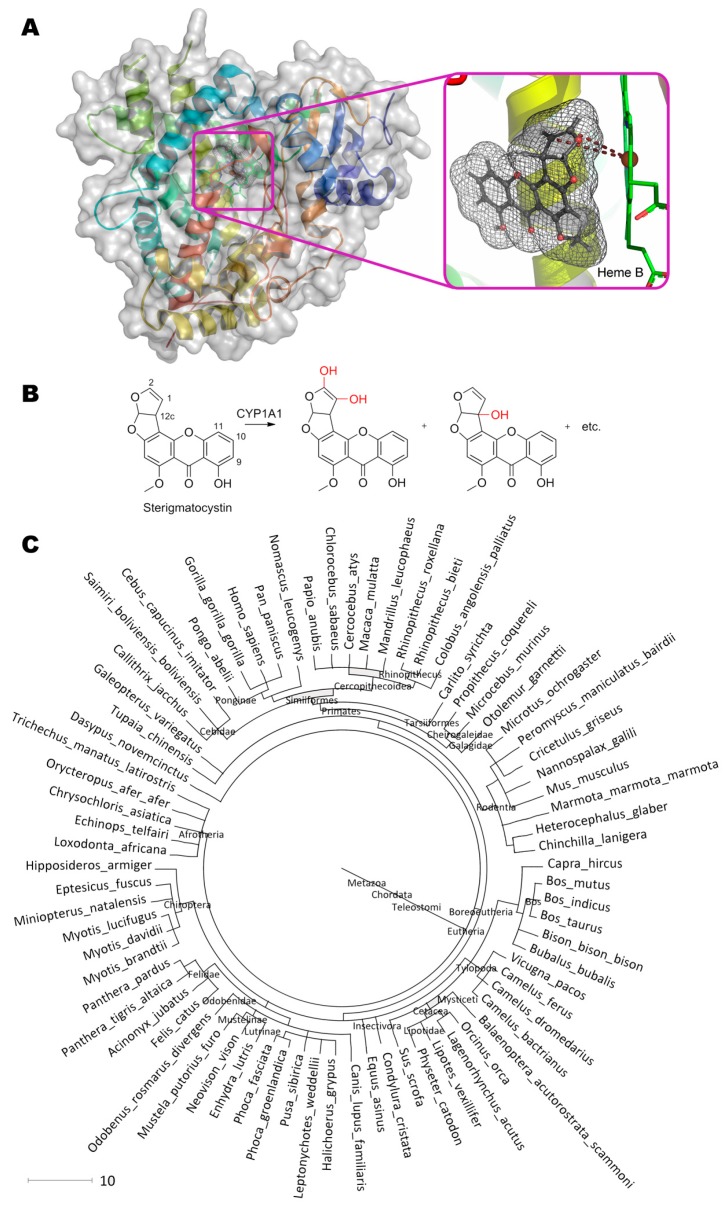
(**A**) Structure of cytochrome P450 (PDB 4i8v) containing sterigmatocystin in its active center, and (**B**) scheme of substrate conversion with cytochrome. Position and geometry of substrate binding was determined as described early. (**C**) Phylogenetic tree of organisms possessing homologous enzymes found with BLAST.

Sterigmatocystin, (3a*R*,12c*S*)-8-hydroxy-6-methoxy-3a,12c-dihydro-7*H*-furo[3′,2′:4,5]furo[2,3-c] xanthen-7-one, being a structural analogue and precursor of aflatoxins, is modified by aforementioned cytochromes at C1 and C2 of the furan cycle (equal to C9 and C8 in aflatoxin B_1_), so as C9, C11, and C12c [85,86]. Molecular docking of such substrate to active center of Cytochrome P450 was in agreement with experimental data (Figure 3). It is interesting that exactly C2 is positioned slightly closer to the Fe(II) (4.5 vs. 5.3 Å), and, therefore, the oxidative attack of the substrate most likely occurs towards it with subsequent formation of the epoxy derivative and its hydrolysis to 1,2-dihydroxy-sterigmatocystin. Besides, other geometries of substrate in the active center are also possible due to absence of additional interactions ensuring the “correct” orientation of sterigmatocystin, for example: When the molecule rotated along the longest axis, an attack on the C12c became available; when orientation of the substrate was “inverted”, C9 and C10 became labile and located from Fe(II) at 4.4 and 5.1 Å, respectively. It should be noted that all possible poses of sterigmatocystin binding within active center were in the same plane both when compared with each other, and when compared with enzyme inhibitor (7,8-benzoflavone), which complexes were obtained experimentally (4i8v).

Thus, an enzymatic activity of cytochrome P450 can be improved towards sterigmatocystin using genetic engineering techniques as in the case of aflatoxin B_1_ [32] by narrowing the cavity of active site and inserting additional amino acids that stabilize the enzyme-substrate complex.

In addition to cytochromes, other mono- or dioxygenases being specific to furofuran moiety, for example, previously mentioned AFO [27], can also modify sterigmatocystin. As compared to aflatoxin B_1_, AFO has a 3-fold greater affinity and a 1.6-fold reduced catalytic constant with sterigmatocystin. Anyway, enzyme activity is rather low and requires significant improvement to be practically sound.

Soybean peroxidase was announced [87] to be able to oxidize sterigmatocystin at pH 5 with *K*_m_ of ca. 1.5 μM. But this value was determined for enzyme immobilized by procedure unreported, and product is unknown. So, someone should use this information carefully.

## 4. Gliotoxin

**Figure 4:**
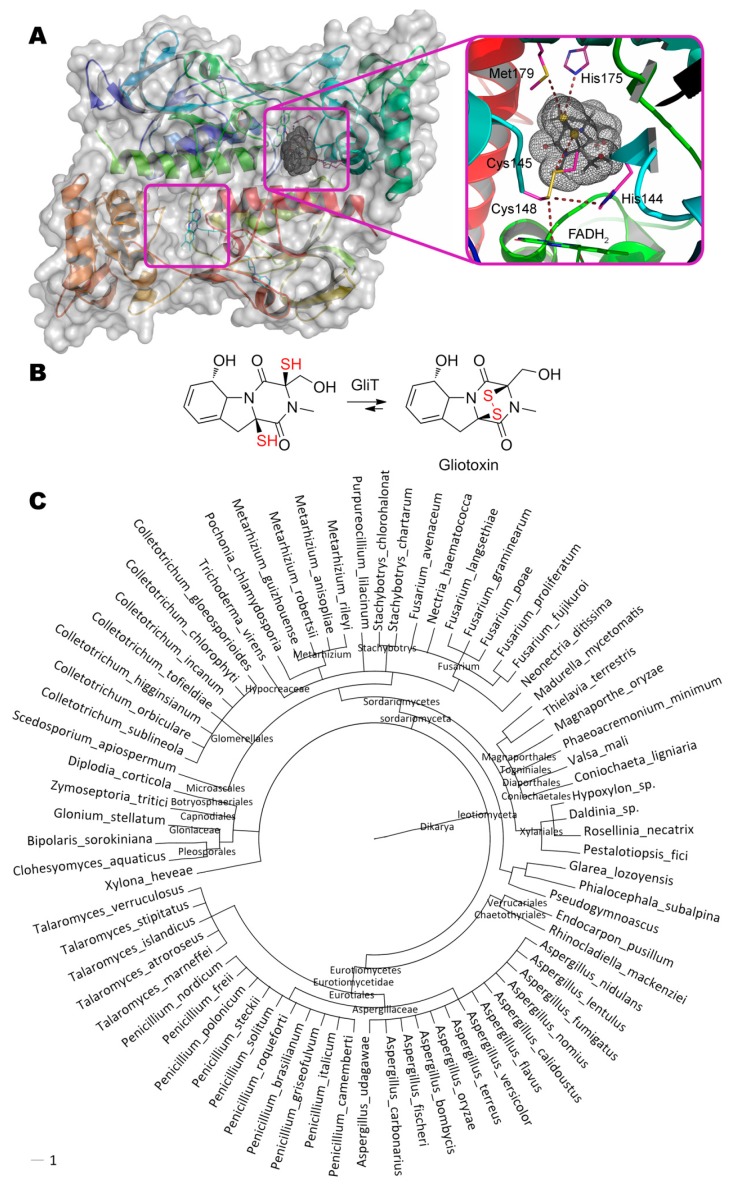
(**A**) Structure of gliotoxin oxidoreductase GliT (PDB 4ntc) containing gliotoxin in its active center, and (**B**) scheme of substrate conversion with GliT. Position and geometry of substrate binding was determined as described early. (**C**) Phylogenetic tree of microorganisms possessing homologous enzymes found with BLAST.

Gliotoxin, (3*R*,6*S*,10a*R*)-6-hydroxy-3-(hydroxymethyl)-2-methyl-2,3,6,10-tetrahydro-5a*H*-3,10a- epidithiopyrazino[1,2-a]indole-1,4-dione, containing a disulfide bridge and synthesized with gliotoxin oxidoreductase GliT (Figure 4) [88], is quite interesting. GliT also contains a disulfide bridge in its active center that is crucial for catalysis. Both experimental data and molecular docking results indicate that within enzyme–substrate complex the catalytic act resembles the movement of swinging spheres in the so-called “Newton’s cradle”: Coenzyme FADH_2_ “strikes” Cys148 (4.0 Å), disulfide bridge Cys148–Cys145 is broken, Cys145 forms a disulfide bridge with the nearest thiol group of the substrate (4.0 Å), and the reverse movement of the second thiol group begins with formation of the disulfide bridge within gliotoxin and restoring the initial Cys148–Cys145 bond of the enzyme.

Enzyme homologues of GliT are widespread among ascomycota’s subdivision *Pezizomycotina* (more than 74 species, including genus *Fusarium*, *Penicillium*, *Aspergillus*) and, therefore, could be useful to develop their version(s) degrading gliotoxin. First of all, residues His175 (3.9 Å) and Met179 (4.3 Å) interacting with thiol group of substrate could be potent in backward reaction resulting in gliotoxin degradation and are the first candidates for rational design. Secondly, microenvironment of Cys145 could be modified to facilitate Cys148–Cys145 bond degradation, or Cys148 could be even deleted at all. Such modifications could also increase actual enzyme activity from the current level of 20 mg/h/mg.

The pathway of gliotoxin biosynthesis is largely reflection to the path of it’s (and other mycotoxins) detoxification in eukaryotes with cytochromes [89] and vice versa.

To restore the oxidized (disulfide) form of glutathione in organisms, glutathione reductase with NADPH cofactor is used. There may be a similar reductase accepting gliotoxin as a substrate by analogy with glutathione (and/or bacillithiol, mycothiol).

On the other hand, dithiol precursor of gliotoxin is formed from a bis-cysteine conjugated derivative with both pyruvate and ammonia being simultaneous co-products [90]. Consequently, some enzymes utilizing pyruvate as a co-substrate may be also useful to modify gliotoxin.

## 5. Zearalenone

**Figure 5:**
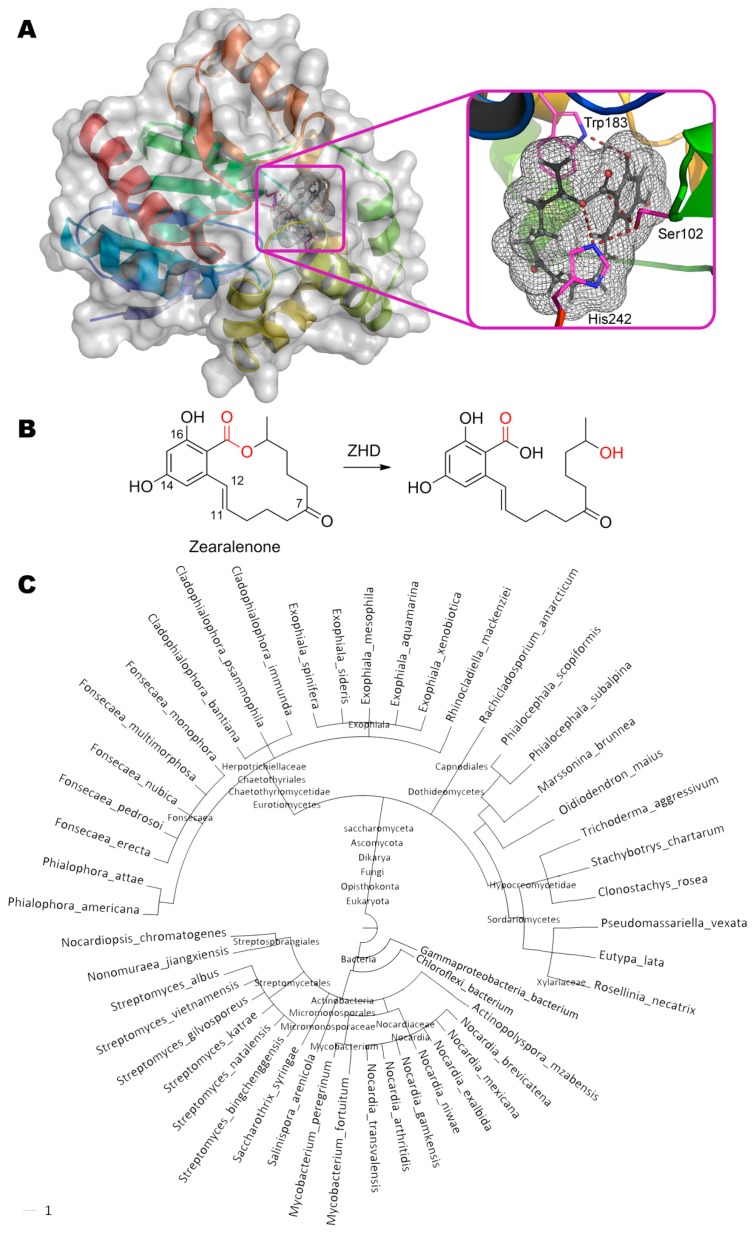
(**A**) Structure of zearalenone hydrolase ZHD (PDB 3wzl) containing zearalenone in its active center, and (**B**) scheme of substrate conversion with ZHD. Position and geometry of substrate binding was determined as described early. (**C**) Phylogenetic tree of microorganisms possessing homologous enzymes found with BLAST.

The degradation of zearalenone ((3*S*,11*E*)-14,16-dihydroxy-3-methyl-3,4,5,6,9,10-hexahydro- 1*H*-2-benzoxacyclotetradecine-1,7(8*H*)-dione) occurs via opening the lactone ring by zearalenone hydrolase ZHD from the fungus *Clonostachys rosea* (Figure 5). This enzyme is actively used by *Clonostachys rosea* for interspecific competition with *Fusarium graminearum* producing zearalenone [91].

The mechanism of the enzyme action is studied in detail by several scientific groups [92,93], and crystal structures of enzyme–substrate and even enzyme–product complexes were solved for enzyme mutants. ZHD having a catalytic triad Ser102, Glu126, and His242 belongs to serine hydrolases [33]. Hydroxy group of Ser102 nucleophilically attack the carbonyl carbon in zearalenone (2.2 Å). According to the hypothesis of authors [92], His242 via Nε forms a hydrogen bond with released hydroxyl group of the product (3.4 Å). But its main function is more likely to increase the nucleophilicity of Ser102 distanced ca. 2.9 Å and/or to finally decarboxylate this residue. Obviously, Glu126 whose carboxyl group is located at 2.9 Å from Nδ of His242, participates in the latter case. In addition to the T-stacking of Trp183 and benzene ring of zearalenone, a hydrogen bond between NH-group and OH-group (2.9 Å) is also possible. According to Gamess-US calculations, the lactone ring of zearalenone is slightly deformed and thus strained as compared to its structure in the ground energy state. Therefore, when the ester bond is broken, this long chain is apparently straightened like a released spring and spreads out from the cavity of the active center (as shown in the PDB 5c8z) [94]. Thereby, removal of the leaving group from the active center is achieved, despite the fact that the product molecule is actually still bound to the enzyme. Perhaps such leaving group elimination as well as wide cavity of the active center is the reason why catalysis is possible at all. For example, acetylcholinesterase possessing exactly the same catalytic triad (Ser203, Glu334, His447) and easily binding this substrate (according to experimental data [95] and our molecular docking), is effectively inhibited by zearalenone, since the long chain cannot leave the active center. By the way, even the massive polycyclic structure of aflatoxin B_1_ can settle within AChE active site (PDB 2xi4), leading to inhibition and allowing it to be used as sensitive element of biosensor for detection of the mycotoxin [96].

On the whole ZHD homologues are widely distributed (more than 50 species of organisms) both among bacteria (including genus *Streptomyces* and *Nocardia*) and fungi.

Rational design followed by site-directed mutagenesis in ZHD was realized in an attempt to improve the specificity of the enzyme towards reduced zearalenone derivatives [97]. Replacement Val153His appears to be the best with the same activity towards zearalenone and 3.7 times increased activity towards α-zearalenol. In this case, *K*_m_ deteriorated by 2.7 times, and the catalytic constant improved by 5.2 times. α-Zearalenol too deeply enters the active center of native ZHD that leads to distortions in the position of His242 being a part of catalytic triad. Due to hydrogen bonding of His153 and OH-group at C7, this substrate is slightly extended from the active center and the correct configuration of the catalytic triad is restored. This is not observed in the case of oppositely directed OH-group of β-zearalenol, and the activity of the mutant enzyme has no improvements.

Homologous enzyme CbZHD from fungus *Cladophialophora bantiana* has more than 5 times greater activity towards zearalenone as compared to α-zearalenol [34]. Moreover, there are several replacements in a number of amino acids surrounding the catalytic triad (by ZHD numbering: Leu132Pro, His134Asn, Met154Ser, Val158Met); as well as a deletion of Val153. It is worth noting that authors [97] also tried to make additional replacements Val158Asp and Val158His, but results were rather negative. What exactly in the naturally modified enzyme caused a decrease in activity (amino acid replacement, or deletion, or both) is not possible to conclude now. However, this example is an excellent illustration of the existence of such “mutant” enzymes in a wild nature.

Another ZHD homologue from fungi *Rhinocladiella mackenziei* possesses 1.8 times more activity towards zearalenone in comparison with α-zearalenol [98]. Apparently, this is due to multiple “natural” genetic mutations (more than 50 of 264 amino acids are affected), including the site mentioned above, by ZHD numbering: Leu131Asn, Leu132Pro, His134Ile, Val153Ala, Met154Asn, Leu155Ser, Asn156Arg, Asp157Ala, Val158Tyr; and deletion of Asn152. An attempt of additional “reverse” replacement Tyr158Phe was more likely neutral (10–20% improvement of activity), and replacement Tyr158Ala reduced activity towards zearalenone by 40% while simultaneously improved activity towards α-zearalenol by 70%. Interestingly, reverse replacement Asn131Leu led to a 3–10-fold decrease of activity towards both zearalenone and α-zearalenol. Single replacement Val153Ala was shown [92,97] to cause a slight decrease of enzyme activity. Obviously, such activity decrease of the ZHD homologue from *R. mackenziei* is compensated by multiple replacements at other sites, which leads to some improvement of activity. Principally, rational protein engineering with site-directed mutagenesis similarly solved such complex problems using compensatory mutations for a long time [99,100].

Proprietary genetically engineered lactonase Zhd518 from *Rhinocladiella mackenziei* [35] has similar mutations near active site as the previous ZHD homologue: Leu131Asn, Leu132Pro, His134Ile, Asn152Ala, Val153Asn, Met154Ser, Leu155Arg, Asp157Ala, Val158Tyr (by ZHD numbering); and deletion of Asn156. Thus, the second changed loop possesses the same sequence Ala–Asn–Ser–Arg–Ala–Tyr as a result. Specific activities decreased in a row zearalenone > α-zearalanol > β-zearalanol ≈ β-zearalenol > α-zearalenol. Replacement Asn153His increased activity towards zearalenone, α-zearalanol, and β-zearalanol by 18–27%, and decreased activity towards β-zearalenol by 20%. An 227% increase of activity towards α-zearalenol was the most profound. What the reason is for such a 2–11 fold difference between Zhd518 and another ZHD homologue from *Rhinocladiella mackenziei* (in favor of the latter) it would be interesting to know. The activity of the enzyme was practically not affected by alkali and alkaline-earth metal ions, as well as EDTA. At the same time heavy metal ions strongly inhibited the enzyme activity (up to 92% with Hg^2+^).

Lactonase ZENC from fungi *Neurospora crassa* [36] is interesting since it is not homologue of ZHD. It is rather homologue of 3-oxoadipate-enol-lactonase (with upto 60% identity, and 73% homologous amino acids), and not 2-(acetamidomethylene) succinate hydrolase as authors suggested (with 23% identity, and 38% homologous amino acids). Anyway, the classical catalytic triad Ser–His–Asp [101] is the same in the both last two lactonases and differs from Ser–His–Glu in ZHD. The typical substrate (3-oxoadipate enol-lactone) is much smaller than zearalenone, and its size and structure is comparable with the same characteristics of patulin. Such 3-oxoadipate-enol-lactonases are used by many microorganisms in the metabolism of cyclic and phenolic compounds [102], and therefore may be widespread. ZENC activity was 1.6 times higher than that of the native ZHD, and therefore a direct comparison of their active center structures could be interesting. Besides, various agricultural raw materials were successfully detoxified using ZENC with 71–95% removal of zearalenone (upto 3.25 mg/kg) for 48 h.

Thermostable peroxiredoxin from *Acinetobacter* sp. [37] and its homologue from *Saccharomyces cerevisiae* [38] are able to detoxify zearalenone. There are several sites in zearalenone for a possible action of peroxiredoxin (e.g., double bond at C11/C12, and hydroxy-groups at C14/C16), but this enzyme is unlikely to act specifically on this substrate and is likely to function as an element of the general protective mechanism of cells (in particular, since its specific activity is modest). Other peroxidases (e.g., from horseradish, rice, etc.) could be even more active by order of magnitude [39]. Possibly, peroxiredoxin homologues were repeatedly identified, but not recognized by a proteomic analysis at the cell level [103]. But their mechanisms should be investigated further.

Zearalenone and its derivatives are known to have different toxicity towards varying species, and the pathways of their metabolism and excretion could be species-dependent [104]. Moreover, a significant contribution to mycotoxin releasing from their glycosylated derivatives is associated with microorganisms in intestine, for example, for zearalenone, its derivatives and trichothecenes [105]. Non-specific β-glycosidase appears to be responsible for that in the case of *Lactobacillus brevis* and *Bifidobacterium adolescentis* [106]. However, according to the same authors, human cytosolic β-glycosidase having a greater specificity for the C14-glycoside derivative can contribute also as in the above-mentioned bacteria. Further metabolism is possible in liver with zearalenone release presumably under the action of carboxylesterases [107].

Glycosyltransferase HvUGT14077 from barley effectively glycosylates zearalenone and its reduced forms (α- and β-zearalenol) at C14 and C16 [40] with 1.5–4.7-fold higher regiospecificity of C14-glycoside derivative synthesis. Following the main substrate exhausting, the same enzyme begins to utilize the mono-derivative with 14,16-diglycoside formation, albeit its activity is much lower (330-430 times) as compared to the best monoglycosylation product. Since HvUGT14077 can glycosylate flavonoids also, they may be its primary substrates. Moreover, HvUGT14077 modify only OH-groups conjugated with benzene ring—in particular, α- and β-zearalenol are not glycosylated at C7; also, another mycotoxin, deoxynivalenol, is not glycosylated at three OH-groups (C3, C7, and C15, see below) which are not associated with a benzene ring. Such selectivity together with varying specificity towards different substrates provokes curiosity in the geometry of these mycotoxins in the enzyme active center but, unfortunately, its structure has not been solved yet.

Similar enzyme UGT73C6 and its analogues with a molar mass of ca. 55 kDa from *Arabidopsis thaliana* catalyze the glycosylation of zearalenone and its derivatives at C14 also [108].

The original plant sulfotransferase SULT from *Arabidopsis thaliana* is proposed for the introduction of sulfo group at C14 of zearalenone [109]. Unfortunately, this enzyme was not developed further due to complications with protein expression and complex cofactor (3′-phosphoadenosine 5′-phosphosulfate) to be recycled, and also due to low enzyme activity towards nonspecific substrate, albeit possible application of transferases of other subclasses to modify zearalenone was shown.

The enzyme from bacteria *Pseudomonas putida* consists two subunits with a molar mass of ca. 60 and 90 kDa and has a maximum activity at pH 7–8 and 30–37 °C [110]. It has the same catalytic activity towards zearalenone and β-zearalenol and slightly reduced towards α-zearalenol. However, further research is needed.

Partially purified enzyme ZDE from *Aspergillus niger* with a molar mass of 50–100 kDa exhibits maximum activity towards zearalenone at pH 4.5 and 40 °C, and was covalently immobilized on rice husk [111]. The product of enzymatic treatment of zearalenone is characterized by reduced toxicity, mutagenicity, oxidative stress and the ability to stimulate apoptosis [112]. However, catalytic mechanism of this enzyme or their complex, as well as the influence of effectors is still unknown, and further studies are required.

## 6. Ochratoxins

**Figure 6:**
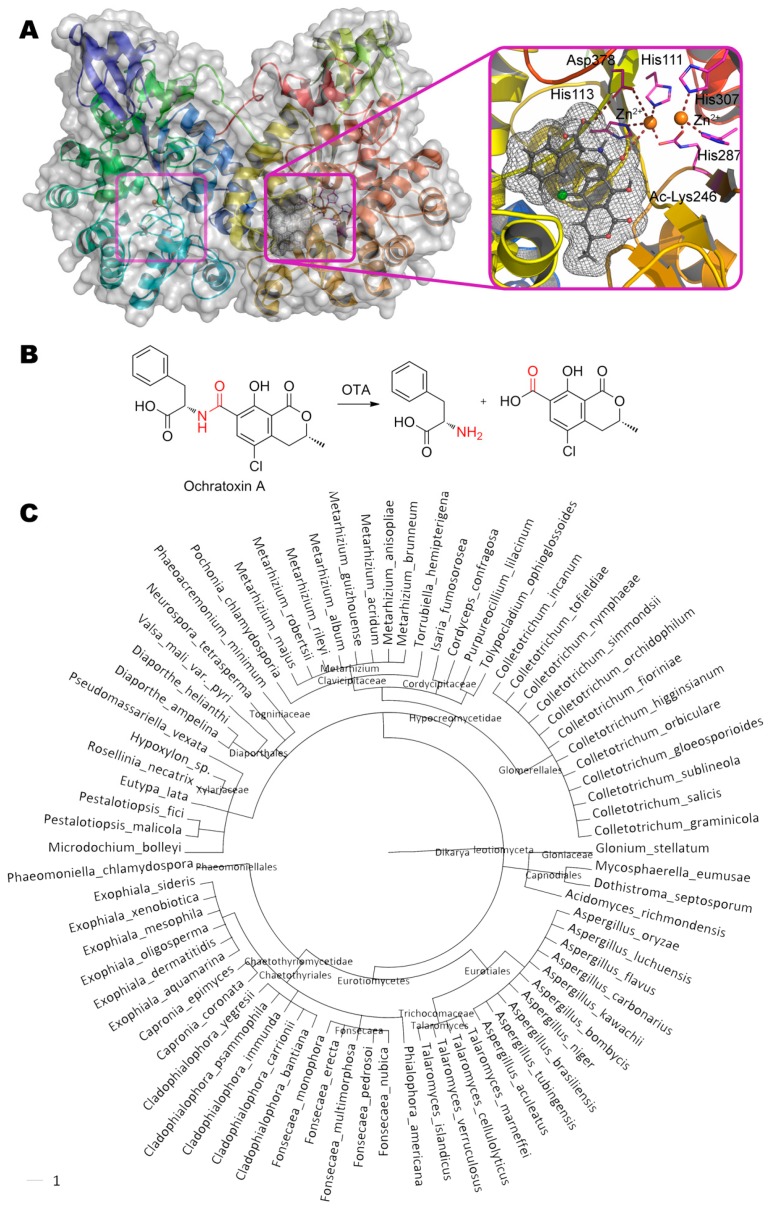
(**A**) Structure of ochratoxinase OTase (PDB 4c5y) containing ochratoxin A in its active center, and (**B**) scheme of substrate conversion with OTase. Position and geometry of substrate binding was determined as described early. (**C**) Phylogenetic tree of microorganisms possessing homologous enzymes found with BLAST.

One of the possible ways of degradation of ochratoxin A (*N*-{[(3*R*)-5-chloro-8-hydroxy-3- methyl-1-oxo-3,4-dihydro-1*H*-isochromen-7-yl]carbonyl}-*L*-phenylalanine) is hydrolysis of its amide bond by ochratoxinase OTase from ascomycota *Aspergillus niger* (Figure 6) [41]. This is a secreted enzyme being sensitive to Zn-specific chelating agents (1,10-phenanthroline) but not inhibited by EDTA. Activity towards ochratoxin B is about 8% of the activity towards ochratoxin A [42].

The active center of this metalloenzyme is classical and widespread in the amidohydrolase superfamily: First Zn^2+^ is coordinated by His287, His307 and two water molecules, and second Zn^2+^ is coordinated by His111, His113, and Asp378. The acylated Lys246 and water molecule act as bridging ligands. As a result, the coordination sphere of the first metal ion has the octahedral geometry, and the second is a trigonal bipyramid. Classically, substrate replaces water molecule from the coordination sphere of one of the metals, and it, thus being enhanced, nucleophilically attacks the reaction center. According to our molecular docking, the nearest metal ion is located at a distance of ca. 3.7 Å from atoms of hydrolyzable bond. In this case, the carboxyl group of phenylalanine can additionally form a hydrogen bond with Asp378 (2.9 Å), and the hydrogen of amide group can interact with His113 (3.1 Å).

Molar mass of OTase homologues (more than 78 fungal species) varies from 39 to 62 kDa. Several such homologues have already been successfully identified experimentally, in particular, in fungi *Aspergillus oryzae*, *Glomerella graminicola*, and *Metarhizium anisopliae* [42]. Thus, there is a great potential for isolation of more active natural variants of this enzyme.

Interestingly, another enzyme hydrolyzing ochratoxin A and isolated from the same *Aspergillus niger* has completely different characteristics as compared to the OTase (Table 1) [43]. In addition to the differences of catalytic characteristics, this enzyme is inhibited by EDTA and not by phenylmethylsulfonyl fluoride (an inhibitor of serine and cysteine hydrolases). Thus, this enzyme is a metal-dependent hydrolase albeit with an unknown metal. Both OTA hydrolase and OTase are more active (up to 600 times) than carboxypeptidase A. Moreover, a number of other serine, cysteine, and metal-dependent proteases can also be used with lower efficiency for hydrolysis of ochratoxin A [113].

Screening among commercially available fungal and bacterial hydrolase preparations allowed to select the most active lipase A from *Aspergillus niger* produced by Amano Corp. [114]. This lipase prefers the same reaction conditions as OTA hydrolase (pH 7.5, 37 °C) and has a molar mass of 32 kDa. Unfortunately, now it is not possible to compare this lipase and abovementioned OTA hydrolase by their specific activity due to the confusion in the definition of activity (viz, added amount of enzyme by definition could hydrolyze all substrate for 1 min).

Although the presence of phenylalanine in ochratoxins makes these substrates acceptable for carboxypeptidase A, a halogen atom (Cl or Br) worsens the catalytic constant of the enzyme by an order of magnitude despite some improvement in *K*_m_ (about 6 times) [115]. At the same time, carboxypeptidase A has a huge number of very close structural and functional analogues for screening of more active enzymes towards ochratoxin A, for example: thermolysin from bacteria *Bacillus thermoproteolyticus rokko* and carboxypeptidase Y from yeast *Saccharomyces cerevisiae* [116], or fungal homologues from *Rhizopus oryzae* and *Trichoderma reesei* [117], or bacterial homologues from *Bacillus amyloliquefaciens* [118], *Acinetobacter* sp. [119], etc. Potentially, other bacteria of the genus *Rhodococcus*, *Pseudomonas* and *Brevibacterium* using ochratoxin A as a source of phenylalanine [120], or *Cupriavidus basilensis* eliminating all the toxicity of this compound [121], can be considered as promising sources of new enzymes to degrade ochratoxins.

## 7. Patulin

**Figure 7:**
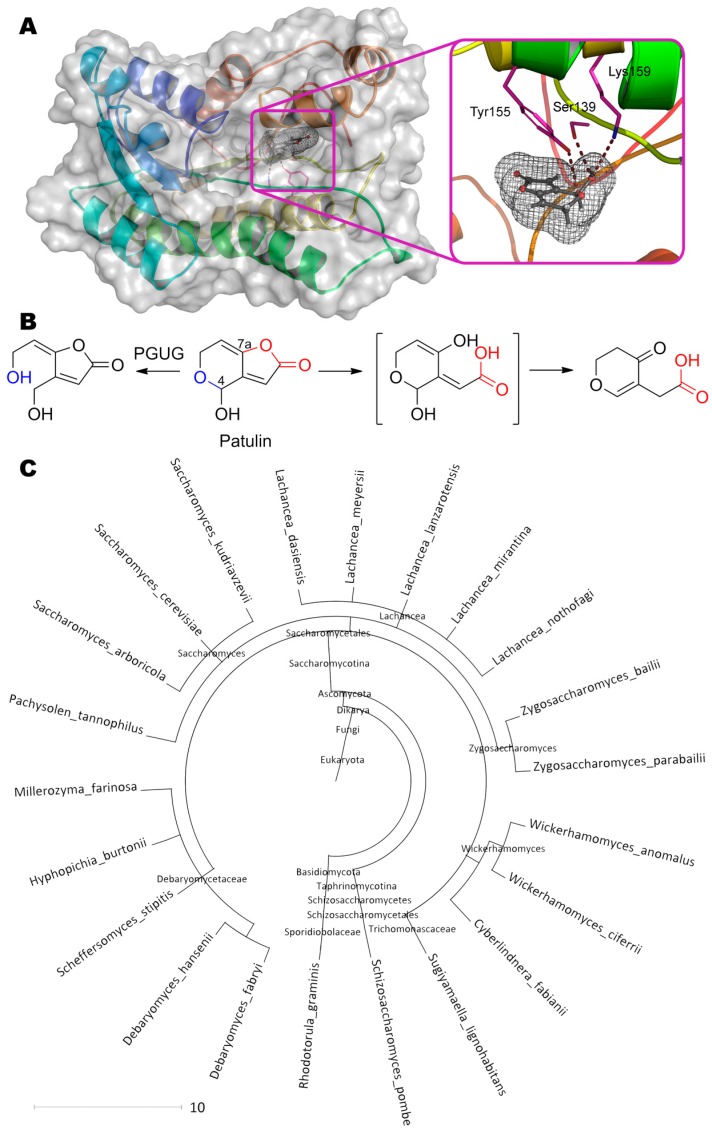
(**A**) Structure of enzyme PGUG containing patulin in its active center, and (**B**) scheme of substrate conversion with PGUG. Amino acid sequence was obtained from GenBank EDK41095.2 and folded using I-TASSER server. Position and geometry of substrate binding was determined as described early. (**C**) Phylogenetic tree of microorganisms possessing homologous enzymes found with BLAST.

Patulin (4-hydroxy-4*H*-furo[3,2-c]pyran-2(6*H*)-one) is perhaps the smallest mycotoxin but it is not less dangerous (Table 1). Today, at least two possible pathways for its detoxification are known and actively investigated. According to the first one [44], the dihydropyrane cycle is opened along the C–O bond at C4 to form (*E*)-ascladiol ((5*E*)-5-(2-hydroxyethylidene)-4-(hydroxymethyl)furan- 2-one) at pH 5 by enzyme PGUG (Figure 7) from yeast *Meyerozyma guilliermondii* (previously known as *Candida guilliermondii*).

Both a predictive analysis of the primary amino acid sequence in the UniProt and a de novo folding followed by a homologue search suggest this enzyme is an oxidoreductase containing NADP^+^ in the active center. The residues Tyr155 (3.4 Å), Ser139 (2.9 Å) and Lys159 (3.8 Å) forming hydrogen bonds with OH-group of pyran and/or with its heterocycle oxygen, can participate in catalysis according to our molecular docking.

Confirming molecular docking data, alignment of PGUG sequence with amino acids of short-chain oxidoreductase Gox2181 from bacteria *Gluconobacter oxydans* [122], which are able to degrade patulin by the same pathway [123], shows their 26% identity and 46% homology. Moreover, amino acids of the active center including the nearest up- and downstream residues, completely coincide: Asn119(Gox2181)↔Asn111(PGUG); Ser147↔Ser139; Tyr162↔Tyr155; Lys166↔Lys159. Gox2181 is an NAD(H)-dependent oxidoreductase, so PGUG could be suggested to use NAD(H) and/or NADP(H) as a coenzyme. At that, Gox2181 catalyses predominantly oxidation and reduction under alkaline and acidic conditions, respectively.

Interestingly, PGUG homologues (more than 22 eukaryotic species) are distributed mainly among yeasts from the *Saccharomycetes* class, thereby allowing them to oppose patulin-producing fungi of genera *Aspergillus*, *Penicillium*, *Byssochlamys*, etc. It is doubly interesting that exactly oxidoreductase is apparently used by these fungi at the last stage of patulin biosynthesis from ascladiol intermediate [124]. However, the current level of enzyme activity has to be substantially improved to make it practically sound.

According to the second path under study [125], the lactone ring is opened at pH 5–6 by enzyme from yeast *Rhodosporidium kratochvilovae*, followed by enol tautomerism of OH-group at C7a (of the original molecule). However, dehydroxylation at C4 occurs simultaneously with inversion of the double bond position. Obviously, some additional yeast enzyme (oxidoreductase) is involved. For example, a similar inversion of the double bond position is observed during isoprenoid biosynthesis by oxidoreductase IspH [126]. Albeit the exact mechanism of such dehydroxylation of patulin is not yet known, the appearance of a similar by-product (4-oxo-4*H*-pyran-3-acetic acid) is observed as a metabolite of grammicin (4-hydroxy-4*H*-furo[2,3-*b*]pyran-2(7a*H*)-one) being an isomer of patulin [127].

**Figure 8:**
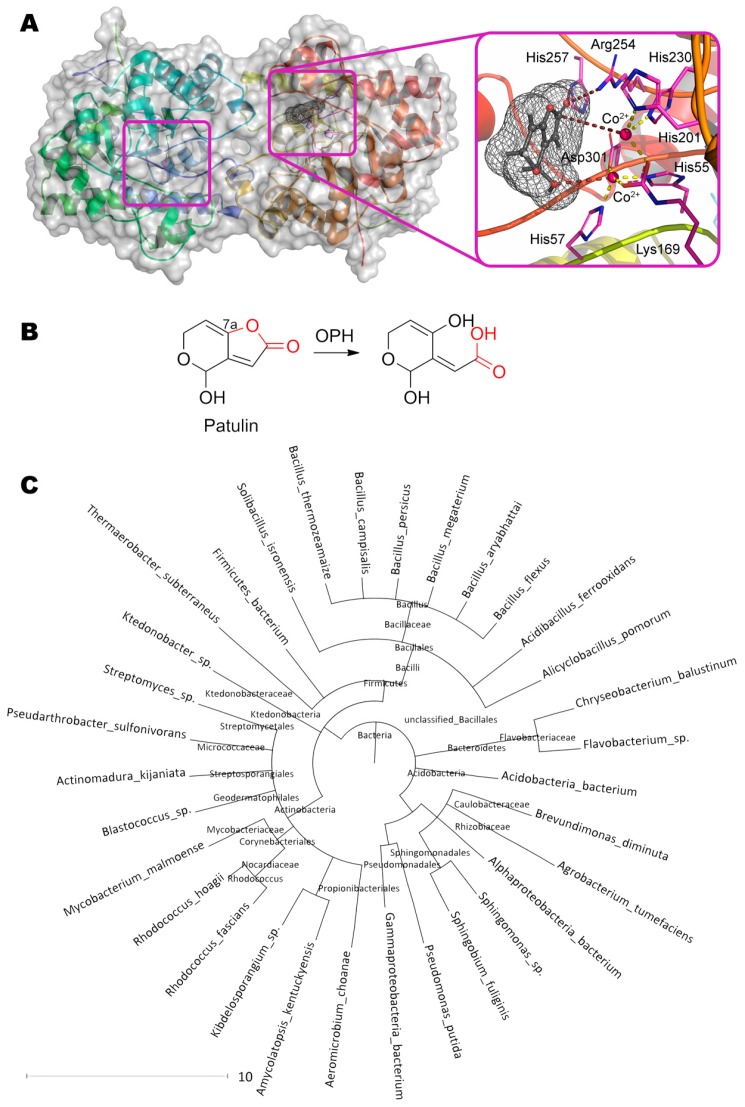
(**A**) Structure of organophosphorus hydrolase (PDB 1qw7) containing patulin in its active center, and (**B**) scheme of substrate conversion with organophosphorus hydrolase. Position and geometry of substrate binding was determined as described early. (**C**) Phylogenetic tree of microorganisms possessing homologous enzymes found with BLAST.

The first stage of the lactone ring opening is also of particular interest. In principle, other enzymes with lactonase activity can have a similar effect. For example, their own lactonases can be in yeast cells [128]. Besides, bacterial organophosphorus hydrolase hydrolyzing *N*-acylhomoserine lactones [129] can theoretically bind patulin (Figure 8). In this case, the first metal ion coordinates the OH-group at C4 of patulin (3.5 Å). The residues His257 (3.2 Å) and Arg254 (3.3 Å) form hydrogen bond(s) with oxygen of the carboxyl group, and the second metal ion (5.3 Å) through the activated water molecule attacks the reaction center in complete accordance with the known enzyme mechanism. Such coordinated action increases activity by several orders of magnitude as compared to PGUG (up to 0.33 mg/h/mg) [130].

Lipase from porcine pancreas was used to obtain biocatalysts hydrolyzing patulin [45,46]. Though mesoporous silica gel SBA-15 had the maximal ad(b)sorption capacity for the enzyme, authors have realized most of the work with another carrier, CaCO_3_. However, the products and the mechanism of such degradation of patulin are not yet known, and possible non-enzymatic decontamination cannot be excluded.

Putative orotate phosphoribosyltransferase from yeast *Rhodotorula mucilaginosa* converts patulin [47]. Naturally this enzyme catalyzes phosphoribosylation of nitrogen within orotate heterocycle with phosphoribosyl pyrophosphate as a co-substrate. OH-group at C4 is the only site to be modified in patulin, resulting in phosphoribosyl-patulin (though used co-substrate was not mentioned in the work). So, (geno)toxicity of the final adduct should be considered since it is quite similar to monophosphates of ribosylated purines.

## 8. Fumonisins

**Figure 9:**
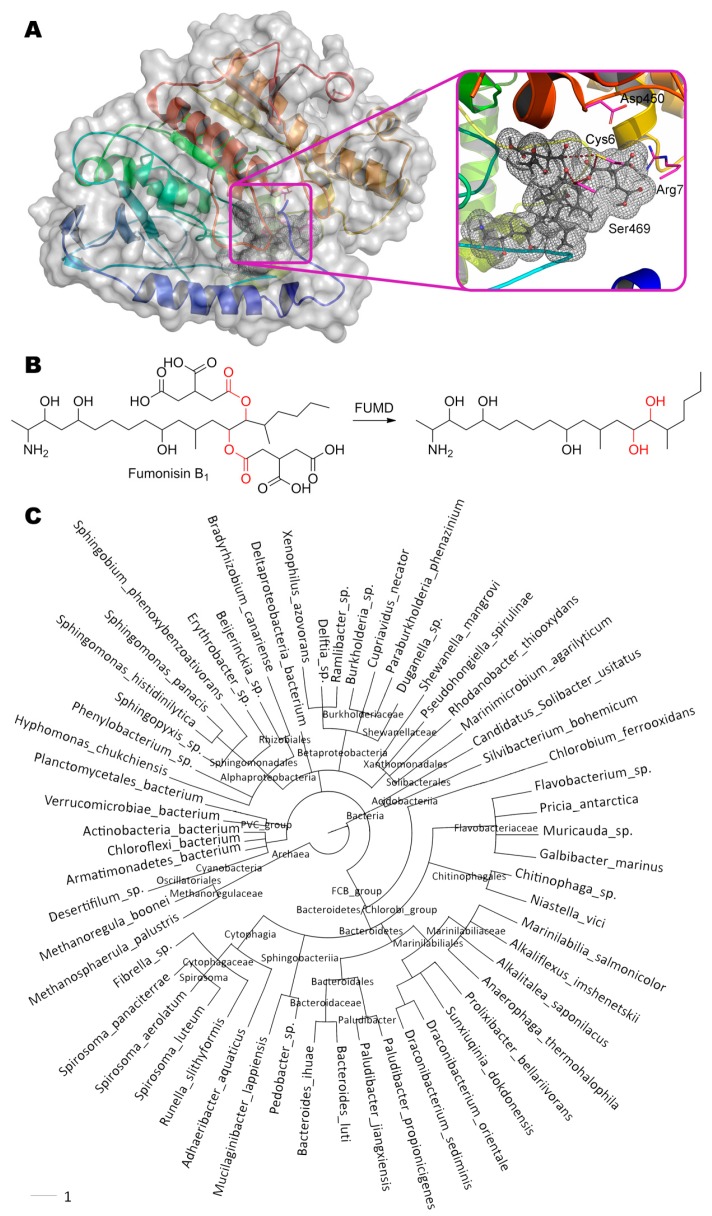
(**A**) Structure of enzyme FUMD containing fumonisin B_1_ in its active center, and (**B**) scheme of substrate conversion with FUMD. Amino acid sequence was obtained from UniProt D2D3B6 and folded as described early. Position and geometry of substrate binding was determined as described early. (**C**) Phylogenetic tree of microorganisms possessing homologous enzymes found with BLAST.

Fumonisin B_1_, (2*S*,2′*S*)-2,2′-{[(5*S*,6*R*,7*R*,9*R*,11*S*,16*R*,18*S*,19*S*)-19-amino-11,16,18-trihydroxy- 5,9-dimethylicosane-6,7-diyl]bis[oxy(2-oxoethane-2,1-diyl)]}disuccinic acid, is hydrolyzed by carboxylesterase FUMD from bacteria *Sphingopyxis* sp. [48] at single or both ester bond while forming one or two molecules of propane-1,2,3-tricarboxylic acid, respectively (Figure 9). FUMD structure is not solved yet as in the case of PGUG, and therefore the same de novo folding and molecular docking was used. Interestingly, Ser469 is approximately equidistant from the both ester bonds (3.0 and 3.9 Å). A similar situation is observed with Cys6 (4.0 and 4.1 Å) which can additionally form a hydrogen bond with Asp450 (3.1 Å) and/or Ser469 (3.4 Å). Thus, catalysis by FUMD can proceed via serine or cysteine hydrolases pathway according to this modeling. Nevertheless, the rate of fumonisin B_1_ hydrolysis is still very low, apparently, due to large size of substrate molecule. However, FUMD homologues are widely distributed among bacteria (more than 61 species), so there is a potential to isolate better version(s). Paranitrobenzyl esterase (pNBE) from bacteria *Bacillus subtilis* is one of these distant homologues [131]. As in the case of PGUG, the entire catalytic triad of amino acids (including their immediate surroundings) completely coincide in FUMD and pNBE despite their 34% identity and 48% homology: Ser189(pNBE)↔Ser240(FUMD); Glu310↔Glu356; His399↔His448. Moreover, these residues are arranged within predicted FUMD structure in the right way (Glu356 – 3.2 Å – His448 – 3.6 Å – Ser240), but are very deep for fumonisin B_1_ access (from 4.8 Å between Ser240 and any ester bond in the substrate). Thereby, fumonisin B_1_ is unlikely to be the main substrate of FUMD.

## 9. Ergot Alkaloids

**Figure 10:**
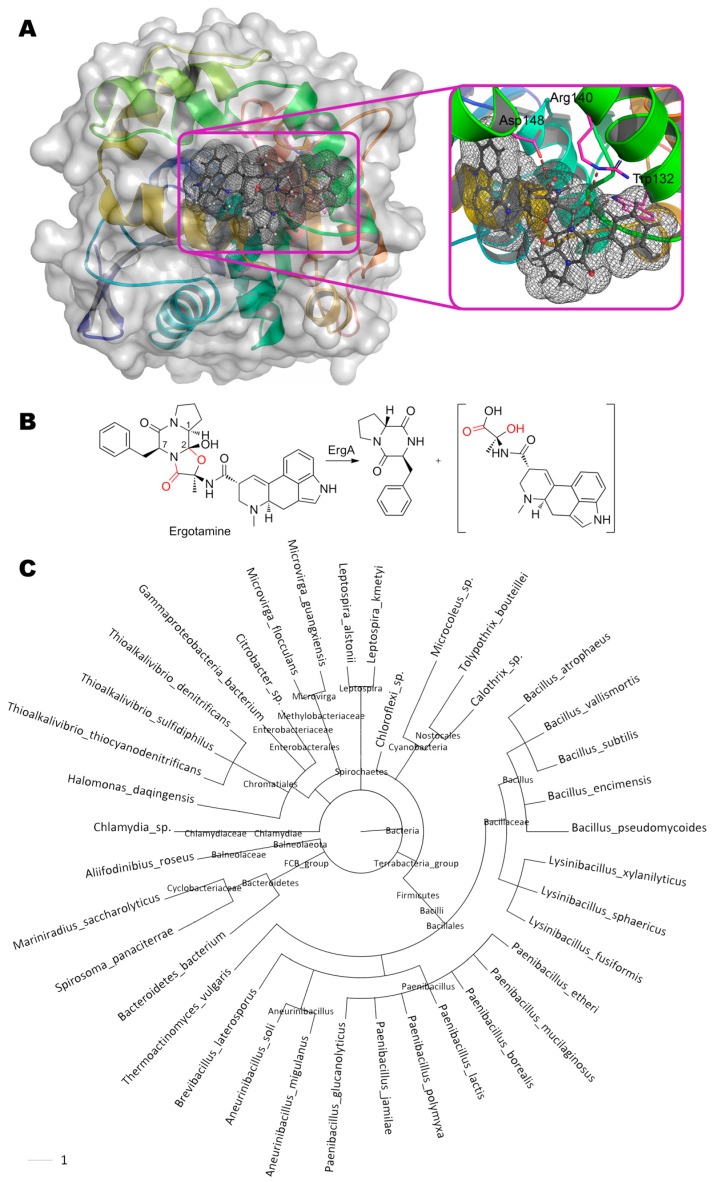
(**A**) Structure of enzyme ErgA containing ergotamine in its active center, and (**B**) scheme of substrate conversion with ErgA. Amino acid sequence was obtained from [132] and folded as described early. Position and geometry of substrate binding was determined as described early. (**C**) Phylogenetic tree of microorganisms possessing homologous enzymes found with BLAST.

Multiple ergot alkaloids are degraded by ErgA from bacteria *Rhodococcus erythropolis* (Figure 10) [49]. Particularly, the products of ergotamine ((6a*R*,9*R*)-*N*-[(1*S*,2*S*,4*R*,7*S*)-7-benzyl-2-hydroxy- 4-methyl-5,8-dioxo-3-oxa-6,9-diazatricyclo[7.3.0.02,6]dodecan-4-yl]-7-methyl-6,6a,8,9-tetrahydro-4*H*-indolo[4,3-fg]quinoline-9-carboxamide) degradation are following: cyclic dipeptide *R*-Pro-*S*-Phe and assumed unstable hemiaminal intermediate, ergine pyruvate.

This intermediate is then spontaneously hydrolyzed to form pyruvate and ergine, which, in turn, is appeared to be deaminated by another enzyme (ErgB) to lysergic acid. The epimerization of carbon at C1 (within initial ergotamine) indicates that nucleophilic or radical attack of the neighboring C2 occurs from the side being opposite to OH-group of this atom relative to the plane of the entire molecule. Whereupon, a proton from C1 is transferred to this nucleophile/radical, followed by proton transfer from OH-group to C1. If ErgA is an oxidoreductase, then action on the amide bond should be accompanied by oxidative hydroxylation of C7 with hemiaminal formation and its subsequent spontaneous hydrolysis to amide and aldehyde [133]. However, this is not observed, and therefore, this enzyme is most likely to be a hydrolase. By analogy to human epoxide hydrolase having both esterase and phosphatase activity [134], it can be assumed that different domains of ErgA are responsible for the hydrolysis of amide and simple ether bonds. Intramolecular decyclization of the furan ring (as in the case of furanose) before or after hydrolysis of the amide bond in it (the reaction is inverse to the first stage of oxazoline biosynthesis [135]) may be an alternative option. As for amide bond, the oxygen of the carboxyl group can be quite close to Arg140 (2.8 Å) and Trp132 (4.4 Å) according to our molecular docking. Though the residue Asp148 cannot directly attack this amide group, it is quite close to hemiaminal group of the proposed intermediate (3.2–3.8 Å) and, therefore, can participate in hydrolysis of this erginopyruvate. As compared to ergotamine, activity towards other substrates is 14% (ergocornine, (5′α)-12′-hydroxy-2′,5′- di(isopropyl)-ergotaman-3′,6′,18-trione), 15% (ergocrystine, (5′α)-12′-hydroxy-2′-isopropyl-5′- (phenylmethyl)-ergotaman-3′,6′,18-trione), 16% (α-ergocryptine, (5′α)-12′-hydroxy-2′-isopropyl-5′- (isopropylmethyl)-ergotaman-3′,6′,18-trione), 50% (α-ergosine, (5′α)-12′-hydroxy-2′-methyl-5′- (isopropylmethyl)-ergotaman-3′,6′,18-trione), and 76% (ergovaline, (5′α)-12′-hydroxy-2′-methyl-5′- isopropyl-ergotaman-3′,6′,18-trione) [132].

Interestingly, a racemate mixture of *R*-Pro-*S*-Phe and *S*-Pro-*S*-Phe is formed in the case of ergotamine degradation by living ErgA producers (*Rhodococcus erythropolis*) and by their biomass disintegrates. That is, there is an enzyme responsible for the isomerization of this dipeptide in the cells. This is understandable, since many cyclic dipeptides (and Pro–Phe too) are used in the Quorum Sensing system in gram-positive bacteria (including *R. erythropolis*). Now it is not possible to distinguish whether this system is specific for ergot alkaloids (to eliminate interference in bacterial cell communication), or it functions normally (and ergot alkaloids are designed to interfere communication). However, ErgA homologues (more than 38 bacterial species) are common among both gram-positive and gram-negative bacteria, and therefore both variants are equivalent.

Sequence alignment of ErgA and distant homologous esterase EaEST from bacteria *Exiguobacterium antarcticum* (24% identity, 41% homology) [136] revealed possible conservative residues of the active site: Ser96(EaEST)↔Ser94(ErgA); Asp220↔Asp234; His248↔His270. Though these amino acids in the predicted ErgA structure are in close proximity (3.6–4.2 Å), they cannot catalyze ergotamine conversion due to closed conformation of this enzyme compartment. The nearest cavity formed predominantly by hydrophobic residues (Val28, Ile168, Trp191, Leu195, Trp210, Phe200, etc.) and being accessible by catalytically active Ser94, is too small to accommodate a hydrolyzable substrate moiety. Moreover, ergotamine entry from solution into this cavity is also sterically impossible. Thus, computer simulation made by us cannot provide an unambiguous answer about ErgA reaction mechanism now, and an experimentally obtained crystal structure of ErgA is required.

## 10. Trichothecenes

**Figure 11:**
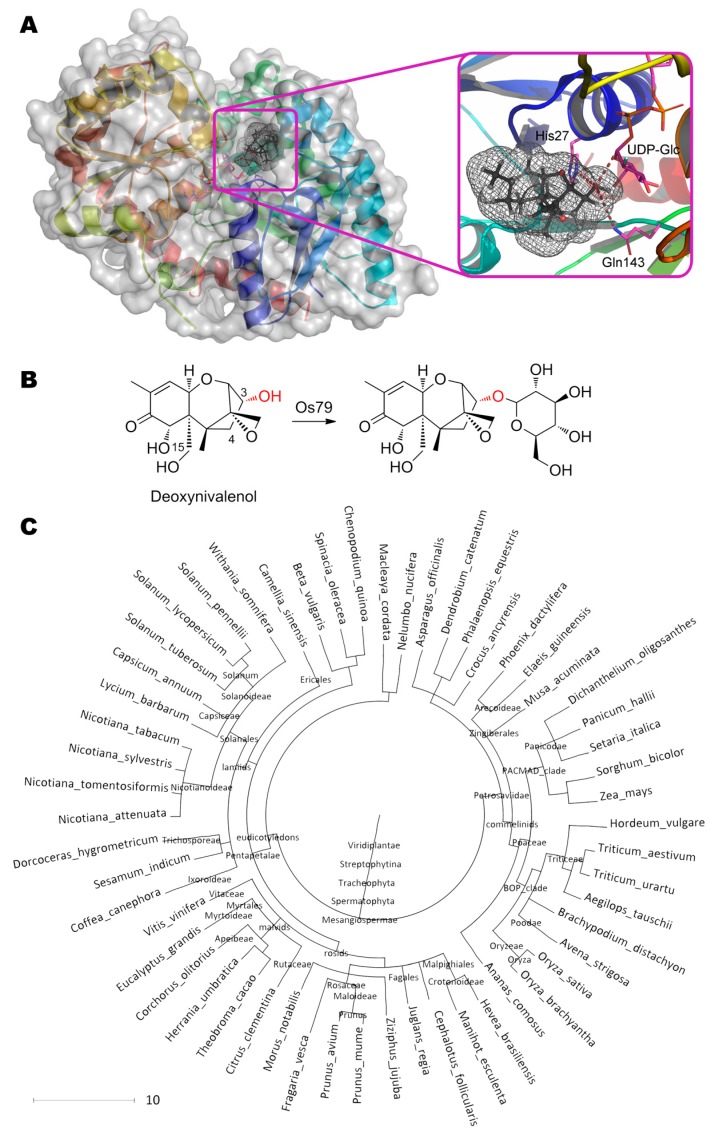
(**A**) Structure of glycosyltransferase OsUGT79 (PDB 5tmd) containing deoxynivalenol in its active center, and (**B**) scheme of substrate conversion with OsUGT79. Position and geometry of substrate binding was determined as described early. (**C**) Phylogenetic tree of microorganisms possessing homologous enzymes found with BLAST.

UDP-glucosyltransferase OsUGT79 from rice is known [50] to transfer glucose from UDP-glucose to OH-group at C3 of trichothecenes (Figure 11). The maximal and minimal activity is observed with deoxynivalenol ((3α,7α)-3,7,15-trihydroxy-12,13-epoxytrichothec-9-en-8-one) and nivalenol ((3α,4β,7α)-3,4,7,15-tetrahydroxy-12,13-epoxytrichothec-9-en-8-one), respectively. The best and worst *K*_m_s are shown towards simplest substrate ((3α)-12,13-epoxytrichothec-9-en-3-ol) and deoxynivalenol, respectively. Catalysis is realized with His27 which is deprotonated by Asp120 (2.2 Å) and which, in turn, pulls the proton away from the OH-group at C3 of substrate (3.6 Å). Thereby, a nucleophilic attack of anomeric carbon in a glycoside becomes possible (3.0 Å). The residue Thr291 (3.5 Å) plays an important role in co-substrate positioning and/or in its deglycosylation. Though it is not discussed by the authors, Gln143 is closely located to substrate/product (3.9 Å) and it may participate in catalytic mechanism. Moreover, replacement Gln143Ala (as well as Phe199Gln) results in a complete loss of enzyme activity according to recent studies of site-directed mutagenesis [137].

Saturation of a genetically engineered enzyme with a co-substrate (UDP-glucose) is observed at 10 mM [51]. In this case, enzyme inhibition by a co-product (UDP) happens at fewer concentrations, and the enzyme has only half of its maximum activity at 1.5 mM UDP. Expectingly, EDTA has virtually no effect on the activity. However, the enzyme is strongly inhibited by the substrate (deoxynivalenol) with *K*_i_ = 104 × *K*_m_ and is half-inactivated at 37 °C for 5–6 h.

Interestingly, very close homologues of OsUGT79 with highly conserved active center are widely distributed among plants (more than 20 species, including TaUGT3 from wheat *Triticum aestivum* [138]). Moreover, the primary substrates of these enzymes such as glycosyltransferase VvGT1 from grape *Vitis vinifera* [139], MtUGT85H2 from *Medicago truncatula* [140], etc. [50], are (iso)flavonoids and other phytohormones, both produced in response to stressful conditions [141]. That is, glycosylation activity of OsUGT79 and its homologues towards trichothecenes is most likely to be collateral.

Another glycosyltransferase AtUGT73C5 from *Arabidopsis thaliana* with a molar mass of 54 kDa [142] capable to glycosylate deoxynivalenol like OsUGT79, is also curious. Possessing 31% identity and 47% homology, and containing the same catalytic dyad (His24, Asp129), the AtUGT73C5, however, cannot glycosylate nivalenol ((3α,4β,7α)-3,4,7,15-tetrahydroxy-12,13-epoxytrichothec-9- en-8-one), which has an additional OH-group at C4 in comparison with deoxynivalenol. Apparently, replacement Thr297Ser (analogue of Thr291 in OsUGT79) and/or Gln152Met (analogue of Gln143 in OsUGT79) could be the reason. The first scenario is supported by the shift of enzyme pH-optimum towards acidic values (p*K*_a_ of Ser side radical is approximately 0.2 less than that of Thr [143]). The second option is argued by the fact that allegedly “natural” substrate of this enzyme is brassinolide ((3a*S*,5*S*,6*R*,7a*R*,7b*S*,9a*S*,10*R*,12a*S*,12b*S*)-10-[(2*S*,3*R*,4*R*,5*S*)-3,4-Dihydroxy-5,6-dimethyl-2-heptanyl]- 5,6-dihydroxy-7a,9a-dimethylhexadecahydro-3H-benzo[c]indeno[5,4-e]oxepin-3-one) [144], which C24 is similar to C4 of nivalenol, and has substituent isopropyl (co-directed to the 4-OH-group of nivalenol) and methyl group, which, thus, are not able to form a hydrogen bond with Gln152.

Neither AtUGT73C5 nor OsUGT79 are able to glycosylate 3-acetoxy-deoxynivalenol. Besides, they are not active towards substrates containing a 4-acetoxy substituent as in the case of T-2 toxin ((2α,3α,4β,8α)-4,15-bis(acetyloxy)-3-hydroxy-12,13-epoxytrichothec-9-en-8-yl 3-methylbutanoate), 4-acetoxy-nivalenol, etc. Therefore, site-directed mutagenesis of OsUGT79 was carried out to increase its hydrophobic pocket [137]. The best activity towards T-2 toxin and diacetoxyscirpenol ((3α,4β)-4,15-bis(acetyloxy)-3-hydroxy-12,13-epoxytrichothec-9-en) was shown by a ternary mutant His122Ala/Leu123Ala/Gln202Ala. However, the authors recommend mutant His122Ala/Leu123Ala/Gln202Leu, as being more balanced towards different substrates. Interestingly, homologous glycosyltransferase HvUGT13248 from barley [52,145] (73% identity and 83% homology to OsUGT79) has native replacements Pro131Ala (analogue of Pro121 in OsUGT79)/Pro134Ala (analogue of Pro124)/Gln210Val (analogue of Gln202), while other key catalytic residues are the same. As a result, both binding and catalytic constant of nivalenol glycosylation are improved ca. 2-fold in spite of some deterioration of catalytic characteristics towards deoxynivalenol. Thus, substrate specificity is changed, and it would be interesting to compare structures of these enzymes for further molecular design of more efficient biocatalysts.

Another cereal glycosyltransferase Bradi5g03300 (70% identity and 80% homology to OsUGT79) has replacements Pro125Ala (analogue of Pro124 in OsUGT79)/Gln203Ala (analogue of Gln202) with the same other residues [52]. Bradi5g03300 is unable to glycosylate 4-acetoxy trichothecenes like OsUGT79, however it has a slightly better affinity and catalytic constant towards deacylated derivatives of T-2 toxin (at C4 and/or C15) like HvUGT13248. As a result, Bradi5g03300 may be 11–14 times more active towards such substrates as compared to deoxynivalenol at substrate concentrations significantly lower than *K*_m_. For example, HvUGT13248 may be 16 times more active towards such deacylated derivatives as compared to deoxynivalenol under similar conditions. Thus, Pro121 in OsUGT79 can play a key role in the inability of OsUGT79 to use 4-acetoxy trichothecenes as substrates, and a single replacement instead of ternary mentioned above may be sufficient to increase the effectiveness of this enzyme.

**Figure 12:**
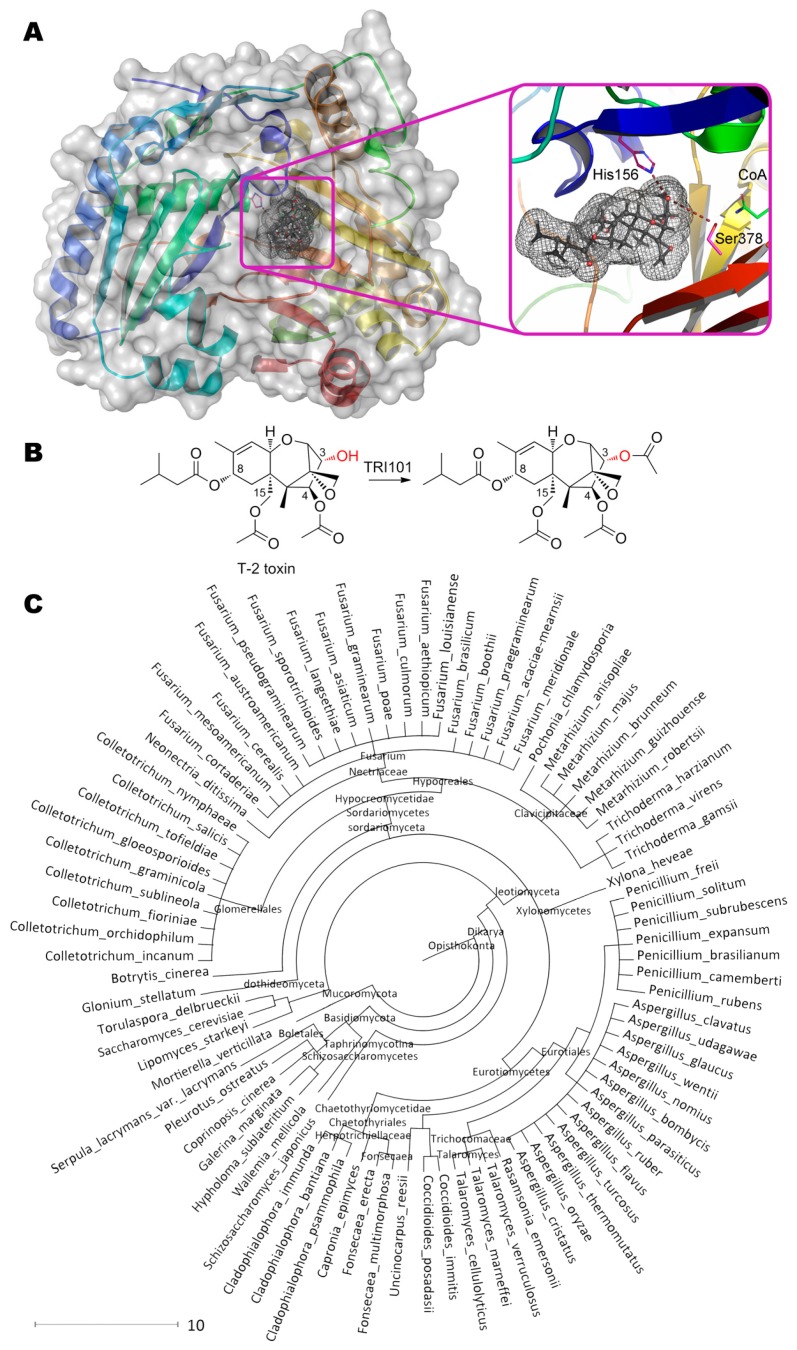
(**A**) Structure of 3-acetyltransferase TRI101 (PDB 2rkv) containing T-2 toxin in its active center, and (**B**) scheme of substrate conversion with TRI101. Position and geometry of substrate binding was determined as described early. (**C**) Phylogenetic tree of microorganisms possessing homologous enzymes found with BLAST.

Interestingly, microorganisms producing trichothecenes have their own 3-acetyltransferases TRI101 utilizing acetyl-CoA as a co-substrate (Figure 12) [53]. According to solved crystallographic structures of several enzymes, the residue His156 participates in catalytic mechanism as a base, deprotonating OH-group of initial substrate and recycling CoA from acetylated intermediate. Enzymes’ specificity significantly varies depending on microorganism strain, species, substrate, etc. [146].

Anyway, the best catalytic characteristics seem to be in the case of FgTRI101 from *Fusarium graminearum* towards isotrichodermol (3α-hydroxy-12,13-epoxytrichothec-9-en) with *K*_m_ and *k*_cat_ being ca. 10 μM and 410 s^−1^ (ca. 7.4 g/h/mg). Yet another issue is investigation of specificity towards other substrates not having 3-OH-group to be modified. More than 80 microorganisms are now known to possess such enzyme(s).

Lipase from fungi *Aspergillus niger* can degrade deoxynivalenol [54]. EDTA has only a limited inactivating effect (<10%), while Zn^2+^ and Cu^2+^ ions significantly inhibit enzyme (up to 60%). Some activation (up to 30%) is observed with Ca^2+^ and Fe^2+^ ions, or with low concentrations of methanol. Based on 18 *m*/*z* increase of the reaction product mass, the authors suggest a mechanism with hydrolytic opening of the epoxy group of deoxynivalenol. Nevertheless, enzyme activity towards deoxynivalenol was 5 orders of magnitude lower as compared to natural substrates, for example, activity towards methyl palmitate was more than 1 g/h/mg.

Aldo-keto reductase AKR18A1 from bacteria *Sphingomonas* sp. catalyzes a reversible oxidation of deoxynivalenol to 3-oxo-deoxynivalenol [55]. The reaction is obviously shifted towards reduction due to higher affinity of the enzyme for 3-oxo-derivative (2.2 times) and activity towards it (6.9 times) compared to deoxynivalenol, as well as greater affinity for NADH (6.2 times) compared to NADP^+^. The inability to use NAD^+^ and NADPH by AKR18A1 is the only obstacle to this.

Asp57, Tyr62, Lys90, and His131 are catalytically active according to amino acid sequence alignment and are typical of such oxidoreductases [147]. According to the classical mechanism, Asp57 coordinates the cofactor and forms a hydrogen bond with Lys90, which, in turn, is able to form a hydrogen bond with Tyr62. His131 is coordinated with oxygen of substrate carbonyl group, thereby making possible to transfer hydride (for reduction) from cofactor to the carbonyl carbon. An uncompensated electron of the carbonyl oxygen is forced to migrate to Tyr62 with transfer of a proton from it and formation of OH-group in deoxynivalenol (for reduction). The stereochemistry of such reaction is maintained with 3*S*-OH-group formation, while there is yet an unidentified enzyme performing an epimerization at this position in the living *Sphingomonas* sp. Interestingly, AKR18A1 can also utilize zearalenone to form α- and β-zearalanol and is present in more than 40 microorganism species.

Oxidoreductase DepA from bacteria *Devosia mutans* was shown to oxidize 3-hydroxy-group of deoxynivalenol [56]. It strongly depends on pyrroloquinoline quinone cofactor, and Ca^2+^ helps to assemble active enzyme form [57].

Different animals have variable sensitivity for deoxynivalenol substitutes, e.g., cytotoxicity increases in a row 3-glucosyl-deoxynivalenol << 3-acetyl-deoxynivalenol < deoxynivalenol ≈ 15-acetyl-deoxynivalenol for porcines [148]. The bioavailability of 3-glucosyl-deoxynivalenol (and therefore sensitivity towards it) is higher for porcines as compared with poults [149]. Partially it could be explained by metabolism pathway through sulfating and glycosylation in chickens and pigs, respectively. From the other hand, porcine microbiota can hydrolyze the 3-glucosyl-deoxynivalenol and release main compound [150]. Human microbiota also can hydrolyze glycosides of deoxynivalenol [102] or other trichothecenes [151], but it will be overestimation that all glycosidases can utilize such glycosides, and actually some enzymes can’t do that [152]. So, thorough selection of active enzyme to unmask such glycosides is required.

Alongside with deoxynivalenol glycosylation there is a deepoxydation pathway in mammals [153]. Though, the enzyme responsible for that is not known currently, there are few candidates like epoxide hydrolases or their homologues with such activity. Anyway, screening of active enzymes is required because their selectivity may vary, e.g., human epoxide hydrolase 1 has no detectable activity towards T-2 and HT-2 toxin [154]. Moreover, direct evolution of bacterial epoxide hydrolase from *Pseudomonas aeruginosa* was attempted [155], but no specifics have followed.

T-2 and HT-2 toxin could be metabolized by porcine CYP3A46 through oxidative hydroxylation of isobutyrate moiety (C3′ atom) [154]. CYP3A4 and other cytochromes are also valid [156]. Though such modification of these trichothecenes seems to be non-relevant for elimination of toxigenic group(s) within toxins, it could be a good stimulus for deeper digging within cytochromes.

Several perspective carboxylesterases were identified in the abovementioned grass *Brachypodium distachyon* [58]. The most promising one appeared to be BdCXE29 which was able to deacetylate trichothecenes at C3, C4, and C15 (except HT-2 toxin having acyl-group at C15). The *K*_m_ was better in 5 times towards 3-acetyl-deoxynivalenol as compared with 15-acetyl-deoxynivalenol. While specific activity (without taking into account a substrate inhibition) was higher in 2 times towards 15-acetyl-deoxynivalenol. That is, a substrate inhibition by 3-acetyl-deoxynivalenol with *K*_i_ of 1.8 mM was revealed. It means that a naïve substrate of this enzyme is larger than 3-acetyl-deoxynivalenol and someone could even try a much more bulky substrate, like ochratoxin A, ergotamine, etc. Interestingly, a lot of homologous carboxylesterases were found in genes of abovementioned *Arabidopsis thaliana*. It was proposed that they are useful for the plant itself as detoxifying agents. However, toxicity of various trichothecenes’ substitutes towards plant can vary significantly during esterification/deesterification [157]. Particularly a glycosylation of trichothecenes could be increased, following the germination [158]. So, balance between plants own needs with issues of toxicity seems to be complicated, if at all possible.

## 11. Multiple Degradation of Mycotoxins

Only a limited number of enzymes are able to modify several mycotoxins simultaneously. There are cytochromes (modifying aflatoxins, sterigmatocystin and trichothecenes), aflatoxin oxidase AFO (acting on aflatoxins and sterigmatocystin), aldo-keto reductase AKR18A1 (reducing zearalenone and trichothecenes) among aforementioned enzymes. That is, the same oxidoreductase can accept various mycotoxins as substrates.

Also, it should be noted that laccase Ery4 from *Pleurotus eryngii* with a molar mass of ca. 58 kDa is able to oxidize various mycotoxins (ochratoxin A, fumonisin B_1_, aflatoxin B_1_, zearalenone and T-2 toxin but not deoxynivalenol) in the presence of enhancing mediators at pH 5 [159]. The most efficient mediators seem to depend on mycotoxin used, and syringaldehyde or (2,2,6,6-tetramethylpiperidin-1-yl)oxyl have shown a maximal efficiency in the most cases. Though some pairwise mycotoxins degradation has been revealed, an enzyme mechanism was not investigated.

Promisingly, multiple strains of *Rhodococcus* cells can degrade both aflatoxin B_1_ and T-2 toxin [160]. A lot of them can utilize zearalenone, and none can convert ochratoxin A and fumonisin B_1_. So, these bacteria could possess active multitarget enzyme(s) detoxifying mycotoxins.

Comparing various enzyme classes (Table 2), it should be concluded that more than a half of enzymes (lyases, isomerases, ligases and translocases) are unable/unknown to act on mycotoxins. While oxidoreductases and hydrolases are the most thoroughly investigated enzymes, the transferases are researched to a lesser degree; they seem to have a great potency to look up. Moreover, there are a lot of mycotoxins (like ergot alkaloids and sterigmatocystin) which currently could be detoxified by a limited number of enzymes only, if it is possible at all.

## 12. Conclusions

Summing up, enzymes have a great biocatalytic potential to solve the problem of mycotoxins’ threat, and several cases of successful commercialization are known [161]. These enzymes realize various mechanisms with these toxic compounds according to published data and our own analysis. Reaction products vary significantly in their structure and toxicity that can be adjusted carefully to minimize or even completely remove possible danger to humans and animals.

The widest set of enzymes acting on mycotoxins and known to date belong to oxidoreductase and hydrolase classes. There are specific and non-specific enzymes: The first ones usually have improved activity, and the second ones can utilize multiple substrate types. Also, such tools against the same mycotoxin seem to have many homologous interspecific analogues from other bacteria, fungi, plants, and/or animals. As a result, frequently it is possible to select enzyme modification with necessary mutation(s) and activity/specificity. Though presently researchers cannot predict activity/specificity with 100% certainty *a priori*, they would implement current advances of rational protein design to obtain quite reliable results.

Molecular docking appeared to be a perfect method to complement crystal structural data for enzymatic mechanism evaluation. Especially it is useful to reveal some tight domains during substrate binding for following rational design.

Fungi are known to produce simultaneously several mycotoxins that should be detoxified. Therefore, complexes of multifunctional enzymes are required in practice. They could be obtained by the combination of some single enzymes, and that can be interesting topic to research.

Enzymes could be combined with other active components also. For example, some secreted biosurfactants like homologue of outer membrane protein A from bacteria *Pantoea* sp. can modulate binding/immobilization of aflatoxin B_1_ [162]. Several mammalian albumins can effectively bind mycotoxins in entropy-driven manner with varying specificity [163]. Enzymes could successfully be combined with pre- and probiotics to treat a feed [164]. Thus, there are a lot of perspective alternatives to be combined with.

Last but not least, the toxicity issue should be mentioned. Though mycotoxins have various modalities of toxic action [2,165,166], currently there are no specific antidotes against their poisoning, since then such enzymes and their combinations can be very powerful and useful tools [167].

## Figures and Tables

**Table 1:** Enzymes detoxifying mycotoxins and their properties. Molar weight (MW) of enzymes corresponds to the value issued in the article or presented in the UniProt database. Optimal or used conditions for determination of enzyme activity are listed. Catalytic characteristics of enzymes are shown towards specified mycotoxin, if not stated otherwise. Tolerable daily intake (TDI)* of mycotoxins is listed for referential purpose and could vary widely depending on the local legislations.

Enzyme (MW)	Source, Ref.	Conditions	Catalytic Characteristics	Comments
**Aflatoxins (aflatoxin B_1_, TDI = 0.11–0.19 ng/d/kg)**				
AKR7A1 with N-His_6_ (37 kDa)	*Rattusnorvegicus* [19]	pH 6.6, 25 °C, 0.2 mM NADPH	*K*_m_ = 21 μM, *V*_max_ = 0.34 μM/min/mg, 6.4 mg/h/mg	–
AKR7A3 with N-His_6_ (37 kDa)	*Homosapiens* [19]	pH 7.4, 25 °C, 0.2 mM NADPH	*K*_m_ = 9 μM, *V*_max_ = 0.6 μM/min/mg, 11.2 mg/h/mg	–
AKR7A5 (38 kDa)	*Musmusculus* [20,21]	pH 6.6, 25 °C, 0.2 mM NADPH	*K*_m_ = 90 μM, *k*_cat_ = 34 min^−1^, 17 mg/h/mg	*K*_m_ = 1.6 μM (NADPH) Inhibition: valproic acid, ethacrynic acid, quercitin, indomethacin
Laccase (56 kDa)	*Trametesversicolor* [22]	pH 4.5, 35 °C	*K*_m_ = 0.28 mM, 0.37 μg/h/mg	–
BADE (22 kDa)	*Bacillusshackletonii* [23]	pH 7–8, 37 °C	0.05 μg/h/mg	Activation: Cu^2+^ Inhibition: Mn^2+^ < Li^+^ ≈ Zn^2+^ < Mg^2+^
MnP (18–42 kDa)	*Pleurotusostreatus* [24]	pH 4–5, 25 °C, 50 μM H_2_O_2_, 50 μM Mn^2+^	up to 2 mg/h/mg	Inhibition: Cd^2+^, Hg^2+^; ≥1 mM H_2_O_2_, Cu^2+^
Peroxidase (32–39 kDa)	*Armoraciarusticana* [25]	pH 7.5, 30 °C, 2.4 mM H_2_O_2_	*K*_m_ = 16 nM, *V*_max_ = 6.4 μM/min, 3–15 μg/h/mg **	–
AFO (77 kDa)	*Armillariella tabescens* [26,27]	pH 5.8–6.0, 30 °C, Cu^2+^	*K*_m_ = 0.334 μM, *k*_cat_ = 2.7 min^−1^, 0.66 mg/h/mg	–
ADTZ (52 kDa)	*Armillariella tabescens* [28,29]	pH 5–8, 35 °C	*K*_m_~ 3 μM **, 0.13 mg/h/mg	Activation: Ba^2+^ Inhibition: Ni^2+^ < Fe^2+/3+^ ≈ Cu^2+^ < Mn^2+^ < Cr^3+^ < Co^2+^ < Zn^2+^ ≈ EDTA
MADE (32 kDa)	*Myxococcusfulvus* [30]	pH 6.0, 35 °C, Mg^2+^	12 μg/h/mg	Activation: Mg^2+^ Inhibition: Li^+^ < Cu^2+^ < Zn^2+^
F_420_H_2_-dependent reductases with N-His_6_ (14–21 kDa)	*Actinomycetales* [31]	pH 7.5, 22 °C, 10 μM F_420_	up to *K*_m_ = 47 μM, *k*_cat_ = 63 min^−1^, 11 mg/h/mg	*K*_m_ = 0.1–0.3 μM (F_420_H_2_)
CYP1A2 with N-His_6_ (59 kDa)	*Homosapiens* [32]	pH 7.4, 37 °C, 1 mM NADPH	*K*_m_ = 30 μM, *k*_cat_ = 0.24 min^−1^, 0.08 mg/h/mg	–
**Sterigmatocystin (TDI = 16 ng/d/kg)**				
AFO (77 kDa)	*Armillariella tabescens* [27]	pH 5.8–6.0, 30 °C, Cu^2+^	*K*_m_ = 0.106 μM, *k*_cat_ = 1.7 min^−1^, 0.44 mg/h/mg	–
**Zearalenone (TDI = 0.5 μg/d/kg)**				
ZHD (29 kDa)	*Clonostachysrosea* [33]	pH 10.5, 30 °C	*K*_m_ = 34 μM, *k*_cat_ = 0.51 s^−1^, 20 mg/h/mg	Inhibition: PMSF, AEBSF
ZHD with N-eGFP (56 kDa)	*Clonostachysrosea* [33]	pH 9.5, 30 °C	*K*_m_ = 10 μM, *k*_cat_ = 0.53 s^−1^, 11 mg/h/mg	–
CbZHD with N-His_6_ (30 kDa)	*Cladophialophora bantiana* [34]	pH 8.0, 35 °C	13 mg/h/mg	–
Zhd518 with N-His_6_ (29 kDa)	*Rhinocladiella mackenziei* [35]	pH 8.0, 40 °C	12.4 mg/h/mg	Activation: Ca^2+^ Inhibition: Li^+^ < Mn^2+^ = Ni^2+^ < Co^2+^ < Cu^2+^ < Hg^2+^
ZENC (30 kDa)	*Neurosporacrassa* [36]	pH 8.0, 45 °C; Na^+^, Ca^2+^, or Mg^2+^	*K*_m_ = 39 μM, 31.8 mg/h/mg	Inhibition: Zn^2+^ < Mn^2+^ < EDTA < SDS ≈ Cu^2+^
Peroxiredoxin (21 kDa)	*Acinetobacter* sp., *Saccharomyces cerevisiae* [37,38]	pH 9.0, 70 °C, 20 mM H_2_O_2_	*K*_m_ = 7.55 mM, 0.14 mg/h/mg	–
Peroxidase (32–36 kDa)	*Armoraciarusticana*, *Oryza sativa* [39]	pH 5–6, 25 °C, 2.4 mM H_2_O_2_	*K*_m_ = 9–40 μM, *V*_max_ = 11–170 nM/min, up to 1.5 mg/h/mg	–
HvUGT14077 (97 kDa)	*Hordeumvulgare* [40]	pH 7.5, 37 °C, 10 mM UDP-G	*K*_m_ = 3 μM, *k*_cat_ = 0.54 s^−1^, 6.4 mg/h/mg	*K*_m_ = 78 μM (UDP-G) Inhibition: UDP
**Ochratoxins (ochratoxin A, TDI = 16 ng/d/kg)**				
OTase (47–51 kDa)	*Aspergillusniger* [41,42]	pH 7.0, 40 °C, Zn^2+^	*K*_m_ = 13 μM, *V*_max_ = 0.29 μM/min, 21.8 mg/h/mg	Inhibition: 1,10- phenanthroline
OTA hydrolase (MW is unknown)	*Aspergillusniger* [43]	pH 7.5, 37 °C	*K*_m_ = 0.5 mM, *V*_max_ = 0.44 μM/min, 2.16 mg/h/mg	Inhibition: EDTA
**Patulin (TDI = 0.4 μg/d/kg)**				
PGUG (27 kDa)	*Meyerozyma guilliermondii* [44]	pH 5.0, 28 °C	0.3 μg/h/mg	–
Lipase (25–64 kDa)	porcine pancreas [45,46]	pH 6.0, 40 °C	0.3 μg/h/mg	–
Putative orotate phosphoribosyl- transferase (25 kDa)	*Rhodotorula mucilaginosa* [47]	pH 4.0, 25 °C	*K*_m_ = 16 μM, *k*_cat_ = 3.4 × 10^−5^ s^−1^, 0.76 mg/h/mg	–
**Fumonisins (fumonisin B_1_, TDI = 2 μg/d/kg)**				
FumD (57 kDa)	*Sphingopyxis* sp. [48]	pH 8.0, 30 °C	3 μg/h/mg	–
**Ergot alkaloids (ergotamine, TDI = 0.6 μg/d/kg)**				
ErgA (35 kDa)	*Rhodococcus erythropolis* [49]	pH 8–9, 35 °C	13 mg/h/mg	–
**Trichothecenes (deoxynivalenol, TDI = 1 μg/d/kg)**				
OsUGT79 (51 kDa)	*Oryzasativa* [50]	pH 8.0, 23 °C, 0.5 mM UDP-G	*K*_m_ = 61 μM, *k*_cat_ = 1.07 s^−1^, 22.4 mg/h/mg	–
OsUGT79 with N-His_6_-MBP (95 kDa)	*Oryzasativa* [51]	pH 7.0, 37 °C, 10 mM UDP-G	*K*_m_ = 0.23 mM, *k*_cat_ = 0.57 s^−1^, 6.4 mg/h/mg	*K*_m_ = 2.2 mM (UDP-G) Activation: Ca^2+^ < Fe^2+^ ≈ Mg^2+^ < Mn^2+^ Inhibition: UDP, DON, Cu^2+^, Zn^2+^
HvUGT13248 with C-His_6_ (53 kDa)	*Hordeumvulgare* [52]	pH 7.0, 25 °C, 1 mM UDP-G	*K*_m_ = 1.5 mM, *V*_max_ = 0.22 μmol/min/mg, 3.91 mg/h/mg	–
Bradi5g03300 with N-MBP and C-His_6_ (96 kDa)	*Brachypodium distachyon* [52]	pH 7.0, 25 °C, 1 mM UDP-G	*K*_m_ = 0.37 mM, *V*_max_ = 19 nmol/min/mg, 0.34 mg/h/mg	–
TRI101 (50 kDa)	*Fusarium sporotrichioides*, *Fusarium graminearum* [53]	pH 8.0, 25 °C, 1.5 mM acetyl-CoA	*K*_m_ = 11.7 μM, *k*_cat_ = 13.5 s^−1^, 288 mg/h/mg	–
Lipase (41 kDa)	*Aspergillusniger* [54]	pH 8.5, 40 °C	4.3 μg/h/mg	Activation: Ca^2+^ < Fe^2+^ ≈ Mg^2+^ Inhibition: EDTA << Zn^2+^ ≈ Cu^2+^
AKR18A1 with His_6_ (37 kDa)	*Sphingomonas* sp. [55]	pH 9.5, 55 °C, 2 mM NADP^+^	*K*_m_ = 1.2 mM, *V*_max_ = 26 nmol/min/mg, 0.46 mg/h/mg	*K*_m_ = 0.48 mM (NADP^+^)
DepA (62 kDa)	*Devosiamutans* [56,57]	pH 7.5, Ca^2+^, 0.1 mM PQQ	*K*_m_ = 32 μM, *k*_cat_ = 4.2 s^−1^, 72 mg/h/mg	Inhibition: Co^2+^ < Cu^2+^ < Fe^2+^
BdCXE29 (38 kDa)	*Brachypodium distachyon* [58]	pH 7.5, 25 °C	*K*_m_ = 0.42 mM (15-ADON), *V*_max_ = 3.4 μmol/min/mg, 69 mg/h/mg	Inhibition: 3-ADON

* Approved TDI or provisional maximal TDI are listed according to Joint FAO/WHO Expert Committee on Food Additives (http://apps.who.int/food-additives-contaminants-jecfa-database/), if not stated otherwise. As being a genotoxicant and carcinogen, aflatoxin B_1_ cannot have an established TDI, so the value is an estimated TDI at a cancer risk level of 10^−5^ (10 per million) in countries where peoples may in addition be exposed to Hepatitis B infection [14]. Sterigmatocystin being carcinogenic too has only provisional dose of low health concern [17] that was specified in the Table. The limit of ergotamine was proposed by European Food Safety Authority [18] and has not been approved yet (as to 2019). Aflatoxin B_1_—(3*S*,7*R*)-11-methoxy-6,8,19-trioxapentacyclo [10.7.0.02,9.03,7.013,17]nonadeca-1,4,9,11,13(17)-pentaene-16,18-dione; sterigmatocystin—(3a*R*,12c*S*)- 8-hydroxy-6-methoxy-3a,12c-dihydro-7*H*-furo[3′,2′:4,5]furo[2,3-c]xanthen-7-one; zearalenone—(4*S*,12*E*)-16,18-dihydroxy-4-methyl-3-oxabicyclo[12.4.0]octadeca-1(18),12,14,16-tetraene-2,8-dione; ochratoxin A—(2*S*)-2-[[(3*R*)-5-chloro-8-hydroxy-3-methyl-1-oxo-3,4-dihydroisochromene- 7-carbonyl]amino]-3-phenylpropanoic acid; patulin—4-hydroxy-4,6-dihydrofuro[3,2-c]pyran-2-one; fumonisin B_1_—(2*R*)-2-[2-[(5*R*,6*R*,7*S*,9*S*,11*R*,16*R*,18*S*,19*S*)-19-amino-6-[(3*R*)-3,4-dicarboxybutanoyl] oxy-11,16,18-trihydroxy-5,9-dimethylicosan-7-yl]oxy-2-oxoethyl]butanedioic acid; ergotamine—(6a*R*,9*R*)-*N*-[(1*S*,2*S*,4*R*,7*S*)-7-benzyl-2-hydroxy-4-methyl-5,8-dioxo-3-oxa-6,9-diazatricyclo[7.3.0.02,6]dodecan-4-yl]-7-methyl-6,6a,8,9-tetrahydro-4*H*-indolo[4,3-fg]quinoline-9-carboxamide; deoxynivalenol—(1*R*,2*R*,3*S*,7*R*,9*R*,10*R*,12*S*)-3,10-dihydroxy-2-(hydroxymethyl)-1,5-dimethylspiro [8-oxatricyclo[7.2.1.02,7]dodec-5-ene-12,2′-oxirane]-4-one. ** The value was calculated using data of referenced authors. Abbreviations: PMSF—phenylmethylsulfonyl fluoride; AEBSF—4-(2-aminoethyl)-benzenesulfonyl fluoride; UDP-G—uridine diphosphate glucose; N-His_6_-MBP—hexahistidine tag and maltose-binding protein on the N-terminus of enzyme molecule; C-His_6_—hexahistidine tag on the C-terminus of enzyme molecule; N-MBP—maltose-binding protein on the N-terminus of enzyme molecule; PQQ—pyrroloquinoline quinone; 3-ADON—3-acetyl deoxynivalenol, 15-ADON—15-acetyl deoxynivalenol.

**Table 2:** Summary of enzyme classes (EC) interesting for use in detoxification of mycotoxins. Potent enzyme classes currently being unknown to modify mycotoxins are marked with query sign (magenta background). Few or multiple enzymes known to posses such activity are designated by single or double plus (cyan background), respectively.

Mycotoxin	EC 1	EC 2	EC 3	EC 4	EC 5	EC 6	EC 7
Aflatoxins	++	?	+				
Sterigmatocystin	+	?	?				
Gliotoxin	?	?	?				
Zearalenone	+	+	++				
Ochratoxins	+	?	++	?			
Patulin	+	+	+				
Fumonisins	+	?	+	?			
Ergot alkaloids	?	?	+				
Trichothecenes	+	++	+	?			

## Appendix A

The structures of mycotoxins for computer modelling were prepared as described previously [168]. Briefly, all compounds were drawn in ChemBioDraw (ver. 12.0, CambridgeSoft, Cambridgeshire, UK). After that, energy minimization was applied using ChemBio3D with force field MM2. Finally, the structures in PDB format were converted to the PDBQT format using AutoDockTools (as part of MGLTools ver. 1.5.6, available at http://mgltools.scripps.edu/) [169] with atomic charges calculated by built-in Gasteiger-Marsili method.

To prepare frameworks of enzymes, known crystallographic structures were treated as described previously for His_6_-OPH [170]. Unknown enzyme structures were predicted using I-TASSER server (ver. 5.1, available at http://zhanglab.ccmb.med.umich.edu/I-TASSER/) [171]. The surface charge distribution of enzymes at their pH-optimum was calculated using adaptive Poisson-Boltzmann solver (APBS, ver. 1.4.2.1) and PDB2PQR (ver. 2.1.1) servers (available at http://www.poissonboltzmann.org/) [172,173] with PARSE forcefield and default settings. Finally, structures in PQR format were converted to the PDBQT format using AutoDockTools.

The mycotoxins were docked to the different enzymes using AutoDock Vina (ver. 1.1.2, available at http://vina.scripps.edu/) [174] on a desktop computer equipped with Intel Pentium Dual-Core CPU E5400 2.7GHz and 3 GB of available memory as described previously for His_6_-OPH [15]. Briefly, the grid box was approximately centered on the active center of the enzyme. The size of the grid box was set up to 500 nm^3^ (8 × 8 × 8 nm). Calculations were performed with default program options, and 12 models (in each run) with the lowest affinities (i.e., binding energy) were selected. Distances and angles between atoms of catalytic groups in the active center of enzyme and ‘labile’ atoms of ligands were measured and visualized with PyMOL Molecular Graphics System (ver. 1.7.6, Schrödinger, LLC, New York, NY, USA).

Molecular surfaces of different mycotoxins were simulated using the GAMESS-US package (ver. 2017 R1, available at https://www.msg.chem.iastate.edu/GAMESS/) [175] under unrestricted Hartree-Fock calculation with hybrid generalized gradient approximations of density functional theory (B3LYP) and basis 6-31G*. Single *p*-type polarization functions on hydrogens under Huzinaga assumption and diffusion of *s* shell to hydrogens were applied. Pipek-Mezey population localization of orbitals was used. To fill up valence, hydrogen atoms were added to the selected mycotoxin models automatically with PyMOL. Calculations were performed with Supercomputer “Lomonosov” of Lomonosov Moscow State University [176] utilizing up to 32 Intel Xeon E5630 2.53GHz, 32 Nvidia Tesla X2070 (Nvidia, Santa Clara, CA, USA) and Intel MPI Library (ver. 5.0.1, Intel, Santa Clara, CA, USA).

Amino acid sequences were aligned with BLAST (available at https://blast.ncbi.nlm.nih.gov/Blast.cgi) [177] at default options. Search for homologues was realized using database of non-redundant protein sequences by *blastp* algorithm. To increase quantity of possible sources, organisms containing the highest rated homologue were iteratively excluded from the search. Finally, all possible sources of selected enzyme were combined and their phylogenetic tree was generated by phyloT (available at https://phylot.biobyte.de) within NCBI taxonomy.
